# Untargeted Metabolomics Reveal Defensome-Related Metabolic Reprogramming in *Sorghum bicolor* against Infection by *Burkholderia andropogonis*

**DOI:** 10.3390/metabo9010008

**Published:** 2019-01-02

**Authors:** Charity R. Mareya, Fidele Tugizimana, Lizelle A. Piater, Ntakadzeni E. Madala, Paul A. Steenkamp, Ian A. Dubery

**Affiliations:** Centre for Plant Metabolomics Research, Department of Biochemistry, University of Johannesburg, Auckland Park 2006, South Africa; charitymareya25@gmail.com (C.R.M.); fideletu@gmail.com (F.T.); lpiater@uj.ac.za (L.A.P.); ntaka.madala@univen.ac.za (N.E.M.); psteenkamp@uj.ac.za (P.A.S.)

**Keywords:** *Burkholderia andropogonis*, defence, flavonoids, metabolomics, phytochemicals, phytohormones, phytoalexins, *Sorghum bicolor*

## Abstract

*Burkholderia andropogonis* is the causal agent of bacterial leaf stripe, one of the three major bacterial diseases affecting *Sorghum bicolor*. However, the biochemical aspects of the pathophysiological host responses are not well understood. An untargeted metabolomics approach was designed to understand molecular mechanisms underlying *S. bicolor*–*B. andropogonis* interactions. At the 4-leaf stage, two sorghum cultivars (NS 5511 and NS 5655) differing in disease tolerance, were infected with *B. andropogonis* and the metabolic changes monitored over time. The NS 5511 cultivar displayed delayed signs of wilting and lesion progression compared to the NS 5655 cultivar, indicative of enhanced resistance. The metabolomics results identified statistically significant metabolites as biomarkers associated with the sorghum defence. These include the phytohormones salicylic acid, jasmonic acid, and zeatin. Moreover, metabolic reprogramming in an array of chemically diverse metabolites that span a wide range of metabolic pathways was associated with the defence response. Signatory biomarkers included aromatic amino acids, shikimic acid, metabolites from the phenylpropanoid and flavonoid pathways, as well as fatty acids. Enhanced synthesis and accumulation of apigenin and derivatives thereof was a prominent feature of the altered metabolomes. The analyses revealed an intricate and dynamic network of the sorghum defence arsenal towards *B. andropogonis* in establishing an enhanced defensive capacity in support of resistance and disease suppression. The results pave the way for future analysis of the biosynthesis of signatory biomarkers and regulation of relevant metabolic pathways in sorghum.

## 1. Introduction

Sorghum [*Sorghum bicolor* (L.) Moench] is the fifth most valuable and highly produced cereal crop that plays an important role in sustainable grain production and food security, particularly in semi-arid and tropical areas. Bacterial leaf stripe disease is one of the three major sorghum bacterial infections considered economically important [1]. The causal agent of the disease is the bacterial pathogen, *Burkholderia andropogonis,* a Gram-negative, motile, aerobic soil bacterium [2,3]. *B. andropogonis* has a diverse and extensive geographical dispersion as well as host range [4]. Bacterial leaf stripe symptoms occur primarily on leaves and are characterised by linear lesions which are red, yellow, tan, or purple in colour, running along the veins due to invasion of the parenchymatous leaf tissue by the pathogen. In some cases the infection not only occurs on the leaves but stems, flower buds, calyxes, peduncle, stalk interior, and seeds [2], destroying plants and reducing crop yields.

Unfortunately, the mechanistic physiological and molecular bases that determine the outcome of the phytopathogenic interaction between sorghum and *B. andropogonis* are not well investigated. Present strategies to aid plants to adapt and defend against such bacterial infection have become alarmingly less effective and some are environmentally unfriendly [5]; hence, new approaches are urgently needed to address important global concerns such as boosting agricultural productivity and sustainable food security [6]. However, to make informed practical decisions on the best tactics in this regard, it is imperative to understand the molecular details of this specific plant‒pathogen interaction at a biochemical level [3,4]. Hence, unravelling the intricacies of the molecular mechanisms underlying the sorghum defensive responses to *B. andropogonis* infection could provide descriptive insights that can be explored for development of improved sorghum cultivars.

In this report, a non-targeted metabolomics approach, based on a liquid chromatography-mass spectrometry (LC-MS) analytical platform, was used to study the biochemical mechanisms involved in the defence response of *Sorghum bicolor* upon *B. andropogonis* infection. Metabolomics is a qualitative and quantitative analysis of the metabolome, used to understand the cellular processes that control the biochemical phenotype of a cell, tissue and whole organism [7]. Newly emerging and maturing metabolomics approaches have proven to be powerful and indispensable tools to interrogate plant biochemistry, thus providing a comprehensive and mechanistic understanding of molecular processes involved in the actual physiological and phenotypic state of the cell or organism under investigation [8,9,10,11,12,13]. 

The reports on interactions between sorghum and bacterial pathogens are limited; hence, there is a poor understanding on the aetiology and epidemiology of these diseases [14]. The current study, therefore, contributes to and expands on this scientific endeavour by profiling defence-related metabolites differentially deployed by sorghum in response to attack by a bacterial pathogen. Such biochemical understanding is vital in the development of new crop protection strategies such as enhancement of natural plant defence mechanisms and production of more resistant cultivars in plant breeding programs.

## 2. Results

### 2.1. Bacterial Leaf Stripe Symptom Progression and Evaluation 

Following treatment of sorghum plants, bacterial leaf stripe progression was visually evaluated by monitoring symptom development on leaves over time. Both NS 5511 and NS 5655 infected leaves revealed typical bacterial leaf stripe symptoms (Figure S1A,B in Supplementary Materials). Small linear tan lesions appeared on the inoculated plant leaves—which progressively elongated with time, nearly encompassing the entire leaf surface, and indicative of the disease progression. The plants started to display symptom development at 3 d.p.i. Appearance of lesions is one of the first visible indicators of host plant–pathogen interactions and in sorghum, pigmented lesions development is regarded as an action set to enhance plant resistance [15]. A disease severity-rating indexing, based on the percentage of leaf area covered in symptoms (Table S1 in Supplementary Materials), indicated that both NS 5511 and NS 5655 cultivars showed similar symptom development, as depicted in Figure S1A,B. However, the symptom progression displayed a cultivar- and time-dependant response to infection. The NS 5655 cultivar (ST) displayed signs of wilting at an earlier stage relative to the former. The NS 5511 (BT) cultivar, thus, appeared to be more resistant to the bacterial infection based on the symptomatology. Although both cultivars are categorised as exhibiting resistance against head smut, leaf disease and stem rot (Capstone Seeds, Howick, South Africa, see Section 4.1), such information was not available with regards to bacterial leaf stripe disease. 

### 2.2. UHPLC-MS Analyses of Sorghum Leaf Extracts

The aqueous-methanol extracts from both infected and non-infected plant leaf tissue samples of NS 5511 and NS 5655 displayed inherent multidimensionality emerging from the complex physicochemical characteristics of the sample constituents. Visual chromatographic inspection showed differential observations which included variation in peak intensities and presence/absence of peaks across samples, indicating differential metabolic profiles: i.e., time-related metabolic responses (Figures S2 and S3) and differential responses between the cultivars (cultivar-related) (Figure S4) to bacterial treatment.

### 2.3. Multivariate Data Analyses: Statistical Description and Explanation of Variation in the Acquired LC-MS Data

**Figure 1:**
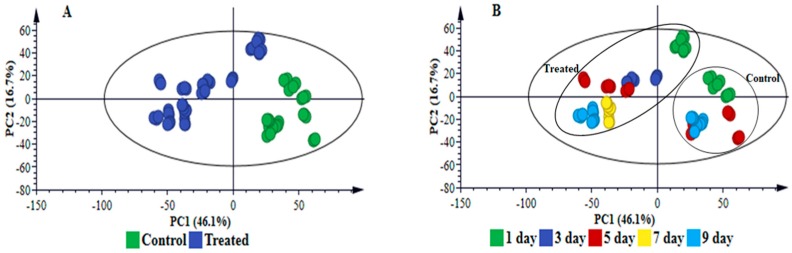
Principal component analyses of the electrospray ionisation (ESI) negative data for sorghum NS 5511 (BT) extracts. (**A**) represents an 11-component model, explaining 84.2% variations in Pareto-scaled data, X, and the amount of predicted variation by the model, according to cross-validation, is 74.1%. (**A**,**B**) represent the same principal component analysis (PCA) scores plot; with (**A**) coloured according to condition (control *vs*. infection treatment) and (**B**) coloured according to time. This two-dimensional scores space, spanned by the first two PCs, reveals infection-related sample clustering (treated/infected = blue, controls = green) and time-related clustering, (days 1–9 post infection) respectively. Note: to determine the group (control/infected) to which the time-related clusters in (**B**) belong, link to the corresponding positions in (**A**).

**Figure 2:**
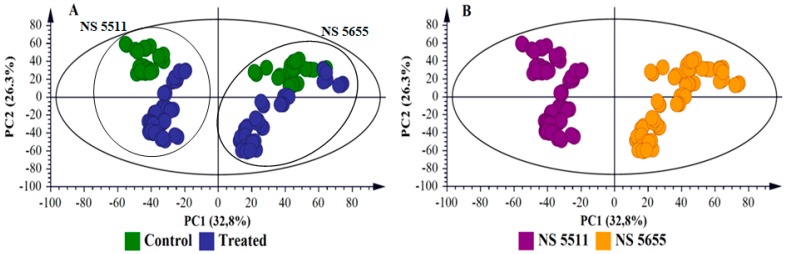
Principal component analyses of the ESI negative data for sorghum NS 5511 (BT) and NS 5655 (ST) extracts. (**A**,**B**) are 15-component models, explaining 86.0% variations in Pareto-scaled data, X, and the amount of predicted variation by the model, according to cross-validation, is 79.6%. (**A**,**B**) represent the same scores plot, with (**A**) coloured according to condition (control, green vs. infection treatment, blue) and (**B**) coloured according to cultivar. This two-dimensional scores space, spanned by the first two PCs, reveals infection-related sample clustering (**A**, control vs. treated) and cultivar-related clustering (**B**, NS 5511 vs. NS 5655).

To further explore and explain differences visually observed in the chromatograms, i.e., to highlight the metabolic changes induced in response to *B. andropogonis* infection, statistical analyses were performed. Multivariate data analysis (MVDA) methods were employed to mine the collected multidimensional data and extract relevant biological information related to the study [8,16]. PCA (Figure 1A, Figures S5A and S6A, Figure 2A and Figure S7A) provided a non-biased reduction of data dimensionality and facilitated the identification of trends and patterns in the data, thus giving an overview thereof [17]. The computed PCA models provided a descriptive visual evaluation of the effect *B. andropogonis* treatment on sorghum plants, revealing time-related sample clustering. This was reflected by the time-trend clusters when the PCA scores plot is coloured based on time points (Figure 1B and Figures S5B and S6B) and cultivar-related sample clustering as shown by separation between the NS 5511and NS 5655 cultivars (Figure 2B and Figure S7B). The clustering of samples reflect the differences in metabolite profiles across the samples and between the two sorghum cultivars respectively. Bacterial infection, thus, induced differential metabolic reprogramming in *S. bicolor* plants, with cultivar-related nuances.

**Figure 3:**
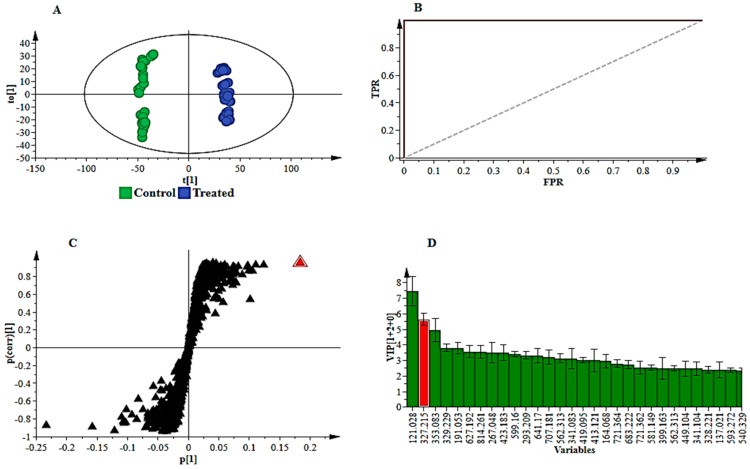
Supervised multivariate analyses of the ESI negative UHPLC-MS data for NS 5511 (BT) cultivar extracts. (**A**): The OPLS-DA score plot shows grouping of control vs. infected for all samples. This model comprises 1 predictive component and 2 orthogonal components (R^2^X= 67.2%, R^2^Y= 99.3% and Q^2^= 98.7%). (**B**): A representative receiver operator characteristic (ROC) plot summarising the performance of OPLS-DA (a binary classification method). (**C**): The OPLS-DA loading S-plot displays the discriminating features (ions) that explain the clustering (sample grouping) observed in the OPLS-DA scores plot, with the features in the top right quadrant positively correlated to the treatment and those in the bottom left quadrant negatively correlated to the treatment. The loading S-plot comprises 1 predictive component explaining 47.1% of the total variation and 2 orthogonal components explaining 20.1% of the total variation. (**D**): A VIP plot summarising the importance of some of the variables in the projection of the model. A VIP value >1 is significant/important in the projection and higher score values indicate an increase in significance of the variables.

To complement the descriptive view provided by PCA modelling, OPLS-DA, a supervised statistical tool, was applied to evaluate and explain the uncovered metabolic changes of sorghum plants responding to *B. andropogonis.* Generated OPLS-DA score plots (Figure 3A and Figure S8A) show evident classification of samples, i.e., the samples are clearly grouped into two distinct classes of treated/infected (blue) and non-treated (green) samples, reflecting the differential metabolite profiles. Furthermore, the OPLS-DA models were validated using various diagnostic tools, to determine how well these binary classification models performed and to also rule out model-overfitting in the supervised modelling [17,18]. These validation steps are regarded as necessary when handling highly dimensional datasets from LC-MS [19]. To confirm the performance of OPLS-DA as a binary classifier, the receiver operator characteristic (ROC) plot was employed. ROC plots (Figure 3B and Figure S8B) graphically summarised a perfect discrimination depicted by the OPLS-DA models (binary classifier) i.e., computed models were perfect classifiers—as revealed by the high sensitivity and specificity (~100%) of the ROC curve [20]. Other additional diagnostic tools used to validate OPLS-DA models are explained in the supplementary file (and displayed in Figures S9A,B and S10A,B).

For extraction of features responsible for the discrimination between infected and non-infected samples, variable selection methods such as the OPLS-DA loading S-plot was used (Figure 3C—NS 5511 and Figure S8C—NS 5655). Significant discriminatory features were extracted for metabolite annotation. The significance of the variables extracted from the S-plot was statistically validated using VIP plots (Figure 3D and Figure S8D). VIP scores evaluation obviated variable selection bias and helped describe the importance of the variables to the model. On the VIP plot, variables with a score of more than 1 are considered significant [16] and an increase in VIP score correlates to increased significance [21]. Therefore, from the VIP plots the variables with a score greater than 1 were chosen for metabolite annotation. Figure 3D (and Figure S8D) show some of the selected variables (e.g., highlighted in red) validated using VIP plots prior to metabolite annotation. Additional tools for further evaluation of selected variables are presented as part of the supplementary data; Figures S9C and S10C, NS 5511 and NS 5655 respectively).

Following validation of the extracted signatory variables considered as important contributors to the class discrimination, metabolite annotation was carried out. These variables relating to metabolic changes following bacterial treatment were annotated to level 2 as classified by the Metabolomics Standard Initiative (MSI): putatively annotated compounds (without chemical reference standards) based on mass spectral information. These metabolites are listed in Table 1. Further details regarding metabolite annotation are given in the experimental section. The fold changes and *p*-values of the various metabolites were obtained from the models constructed from all control samples against all the infected samples of the NS 5511 and NS 5655 cultivars.

**Figure 4:**
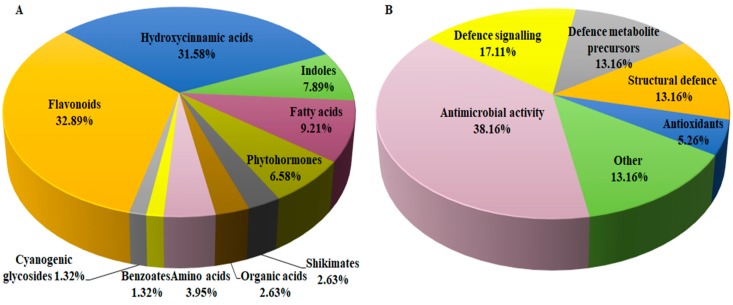
Classification of the annotated signatory / discriminatory metabolites in extracts of sorghum plants responding to infection by *B. andropogonis*, according to the chemical classes (**A**; see Table 1) and primary/prominent functions in defence (**B**; see Section 3.1, Section 3.2, Section 3.3 and Section 3.4 for functions of some annotated metabolites). A total of 76 plant metabolites were putatively identified. (**A**): Illustrates the chemical diversity of metabolites potentially contributing to defence against *B. andropogonis* (flavonoids and hydroxycinnamic acids—two major classes). (**B**): Due to some metabolites possessing more than one function, grouping was based on the known primary/prominent role in plant defence.

The visual inspection of symptoms and chromatographic analysis results, further investigated using various statistical tools, evidently pointed to metabolic reprogramming in sorghum plants responding to *B. andropogonis* infection. As seen in Table 1 and summarised in Figure 4A, LC-MS-based untargeted metabolomics facilitated annotation and analysis of an array of chemically diverse metabolites, representing a wide range of metabolic pathways associated with the plant defence response. Chemical classification (Figure 4A) highlighted two major classes i.e., flavonoids and hydroxycinnamic acids (HCAs), highlighting the significance of these metabolites in sorghum defences. In addition, the annotated metabolites demonstrated to possess various defence-related functions as summarised in Figure 4B. For example, challenged sorghum plants activated both structural and chemical defences to counteract pathogen infection. The diversity of the metabolites, arising from different metabolic pathways (particularly amino acid, fatty acid, shikimic acid, phenylpropanoid and flavonoid metabolic pathways), showed an intricate and dynamic network of the sorghum defence arsenal towards *B. andropogonis* in resistance and disease suppression. Moreover, it correlates with what is known about cereal responses towards pathogen attack [22] and reflects the significant genetic diversity and extensive adaptive abilities of sorghum [23]. The metabolic fluctuations in response to bacterial treatment as well as the functional roles of the annotated metabolites in defence responses are detailed in the following section. Relative quantities, expressed as fold changes of the putatively identified metabolites, were used to provide measurable evaluation of metabolic changes and to give a comprehensive picture of the reprogramming of *S. bicolor* metabolism.

## 3. Discussion

### 3.1. Metabolic Reprogramming of Primary and Secondary Metabolism—The Role of Aromatic Amino Acids in Pathogen-Induced Stress Responses

Infection of sorghum with *B. andropogonis* triggered changes in amino acid metabolism. These alterations occurred in the three aromatic amino acids, l-tyrosine, l-tryptophan and l-phenylalanine. Relative quantitative analyses revealed a decrease of these amino acids in the respective levels (fold change < 1; Table 1) in infected plants over time (time-related changes; 1–9 d.p.i.) as well as cultivar-related differences in metabolite levels. The role of metabolic pathways of distinct amino acids in the regulation of defence responses in pathogen-challenged plants has been demonstrated in several studies [24,25,26]. Aromatic amino acids synthesised via the chorismic—shikimic acid pathway are central to plant metabolism—serving as precursors in the synthesis of a range of secondary metabolites and phytohormones with plant defence functions [27,28].

Phe is an important precursor for phenylpropanoid and flavonoid pathways, whereas Tyr and Trp are involved in cyanogenic glycosides and indole metabolite synthesis respectively [27,29]. Additionally, Phe is a precursor in synthesis of signalling molecules such as the phytohormone salicylic acid (SA), pivotal in launching of plant defence. Thus, the decrease in levels of the amino acids and increase in the levels of some of the downstream derivatives following infection, suggests the channelling of these precursors into metabolic pathways of Phe-derived (e.g., phenylpropanoids, flavonoids and SA), Trp-derived (e.g., indole and serotonin derivatives) and Tyr-derived defence-related metabolites (e.g., dhurrin) [27,30]. 

### 3.2. Differential Changes in Fatty Acid Metabolism

**Figure 5:**
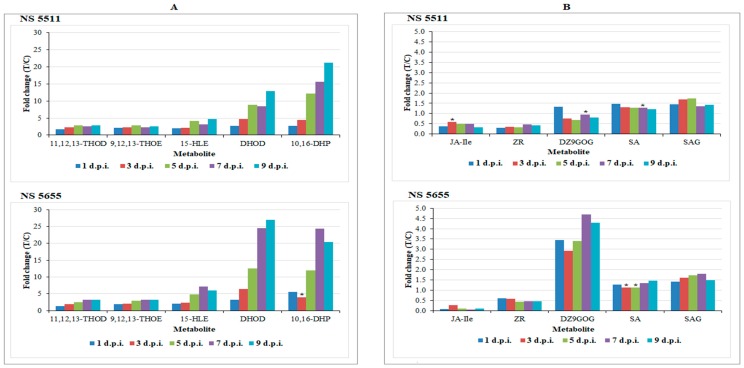
Relative quantification of fatty acids (**A**) and phytohormones (**B**) annotated in sorghum leaves responding to infection by *B. andropogonis*. The relative levels of each metabolite are expressed as fold changes, computed from treated against control (T/C) samples, where fold change > 1 represents significant accumulation in NS 5511 (BT) and NS 5655 (ST). Abbreviations: (A) 11,12,13-THOD = 11,12,13-trihydroxy-9,15-octadecadienoic acid; 9,12,13-THOE = 9,12,13-trihydroxy-10-octadecenoic acid; 15-HLE = 15-hydroxylinoleic acid; DHOD = dihydroxy-octadecadienoic acid; 10,16-DHP = 10,16-dihydroxypalmitate (B) JA-Ile = jasmonoyl-l-isoleucine; ZR = zeatin riboside; DZ9GOG = dihydrozeatin-9-*N*-glucoside-*O*-glucoside; SA = salicylic acid; SAG = salicylic acid 2-*O*-beta-d-glucoside. The fold changes on the various treatment days presented in the graphs have a *p*-value < 0.005, except for the ones indicated by * with *p*-value > 0.005, but < than 0.05.

The results reveal the significant accumulation of fatty acids in *B. andropogonis*-challenged plants (Table 1), and the levels of 15-hydroxylinoleic acid, 10,16-dihydroxypalmitate, dihydroxy-octadecadienoic acid, 11,12,13-trihydroxy-9,15-octadecadienoic acid and 9,12,13-trihydroxy-10-octadecenoic acid were found to considerably increase over time (Figure 5A) in both cultivars. The increase in free fatty acid levels (particularly unsaturated) has been reported in pathogen-stressed plants ‒ linoleic (18:2), and linolenic acids (18:3) have been linked to increased resistance to *Colletotrichum gloeosporioides* in avocado and *Pseudomonas syringae* in tomato [31] respectively. Mutant *Arabidopsis* plants compromised in production of trienoic fatty acids have been shown to be susceptible to *P. syringae* [32].

Compelling evidence from several studies have demonstrated induced activation of NADPH oxidase by linoleic- and linolenic acid, leading to production of ROS [31,32,33]. Accumulation of the latter can result in the fragmentation/cleavage of fatty acids into various products that can act as chemical inducers of defence responses. In *Arabidopsis* as well as other plants, azelaic acid (a cleavage product) acts as an inducer of SAR via the accumulation of SA [31,32]. Additionally, cell death-inducing activity of some fatty acids has been reported [34].

The hydroxy fatty acids, 15-hydroxylinoleic acid (avenoleic acid), 9,12,13-trihydroxy-10-octadecenoic acid (pinellic acid) and others annotated in Table 1, are classified as oxylipins, synthesised from linoleic acid in cereal crops and in other plants [35,36]. 9,12,13-Trihydroxy-10-octadecenoic acid together with other trihydroxyoctadecenoates were reported to be produced in response to fungal infection, conferring resistance to a spectrum of fungal pathogens. Previous reports have also highlighted the growth-inhibitory roles of trihydroxy derivatives of linoleic- and linolenic acids to plant fungal pathogens [36,37]. Generally, oxylipins perform defence roles in plant innate immunity as signalling molecules; inducing defence responses or as compounds exhibiting antimicrobial properties [33,38]. Antimicrobial activities of epoxy- and hydroxy-fatty acids and other oxylipins towards bacterial pathogens, and growth inhibition properties to a spectrum of pathogens were reported [38]. 

In local defence, fatty acids (particularly C16 and C18) are involved in the formation of cutin in the plant cuticle that confers resistance against pathogens by hindering pathogen ingress and proliferation in the host [31,33]. The synthesis of 10,16-dihydroxypalmitate and 16-hydroxypalmitate (major cutin monomers) annotated in Table 1 can be interpreted as an attempt to strengthen the cuticle and limit further bacterial ingress [39].

Apart from acting as hydrophobic hormones in modulating signal transduction pathways and antimicrobial compounds, fatty acids also serve as precursors to the phytohormone jasmonic acid (JA) [32,38] and as essential constituents of membrane lipids in plants. The up-regulation of the annotated fatty acids of known function thus supports a functional role in sorghum defence/resistance [31,32,33].

### 3.3. Plant Hormones: Regulatory and Signalling Molecules in Sorghum Defence Responses

The intricate defence responses of sorghum also involved several phytohormones such as SA and SA glucoside, JA and zeatin derivatives (Table 1). Drawing attention to the well-known plant stress phytohormone, SA and its conjugate (salicylic acid 2-O-beta-D-glucoside (SAG), augmented levels were noticed following bacterial treatment (Figure 5B). SA levels in NS 5511 (BT) were elevated in the early stages of bacterial infection (1–3 d.p.i.) as compared to NS 5655 (ST), which showed increased levels in the late stages (5–9 d.p.i.). This finding postulates an early onset of defence responses in NS 5511 compared to NS 5655, and corroborates the view that the former cultivar is more resistant than the latter. However, for SAG the levels between the two cultivars were comparable. Plant hormones play various biological roles, including signalling in stress responses, inducing secondary metabolite accumulation [40,41]. Experimental evidence has shown a link between the accumulation of SA and SAG, expression of PR proteins, accumulation of phenylpropanoids and resistance to pathogens in a range of plants [21,24]. 

SA, a phenolic phytohormone synthesised from cinnamic acid or isochorismate [24,31] plays a key role in local and systemic defence [42]. This hormone accumulates in regions around the infection site, stimulating the hypersensitive response (HR) to result in necrotic lesions that limits pathogen proliferation. The HR can be linked to the observed development of lesions on *B. andropogonis*-infected sorghum plants (Figure S1A,B) [43]. Moreover, SA is a major signalling molecule triggering SAR, leading to up-regulation of PR proteins and enhancement of phenylpropanoid accumulation [26,44]. The production of and signalling function of SA, is highly important in plant immunity towards pathogens exhibiting biotrophic and hemibiotrophic lifestyles. The detection of SA and SAG as signatory biomarkers, therefore, indicates their importance in triggering defences against *B. andropogonis* [26,45]. 

The highly biologically active derivative of JA, jasmonoyl-l-isoleucine (JA-Ileu), is a known modulator of stress responses [46]. Although this phytohormone was identified to be present, it was not statistically significant and not significantly related to the treatment, as indicated by the very low levels detected upon quantitative evaluation (Table 1 and Figure 5B). Jasmonates derived from linolenic acid metabolism are also classified as oxylipins. These molecules are involved as signalling molecules in activation of defence responses towards both abiotic and biotic stressors [31,41]. Exogenous application of JA results in defence-related gene expression as well as production of antimicrobial compounds [33,41]. The accumulation of this phytohormone is mostly associated with necrotrophic pathogens and also triggers ISR [47].

The levels of two zeatin conjugates, dihydrozeatin-9-*N*-glucoside-*O*-glucoside and zeatin riboside, were also found to be altered as part of the induced host responses (Figure 5B). The levels of the former were dramatically higher in NS 5655 as compared to NS 5511 (Figure 5B). Zeatin and derivatives thereof (e.g., riboside and glucosides) are regarded as the principal group of isoprenoid cytokinins (CKs) in plants. Previously, *trans*-zeatin cytokinins were demonstrated as the more active class in enhancing resistance against pathogens; however, recently, the role of *cis*-zeatin CKs in regulating plant defence responses and as ‘novel’ stress-response markers has been highlighted [48,49]. CKs can act synergistically with SA in the activation of defence gene expression [50] and enhancing of resistance by zeatin CKs to be linked to an increase in cell membrane integrity [49,51]. The identification of the various phytohormones revealed an interplay of hormonal activity in sorghum defence signalling and regulation. Interaction (antagonistically or synergistically) and fine-tuning between phytohormones governs activation of a range of defences, including those specific to the stressor [41,47,50]. The identification of phytohormones as biomarkers can, therefore, be explained as triggers of defence responses to *B. andropogonis* infection.

### 3.4. Metabolic Reprogramming of Defence-Related Metabolites Derived from Shikimic Acid-, Phenylpropanoid- and Flavonoid Pathways 

The metabolic reprogramming in sorghum following *B. andropogonis* infection involved perturbations in the pool of metabolites synthesised via the shikimic acid-, phenylpropanoid- and flavonoid biosynthetic pathways—which are partially interlinked [52]. The shikimic acid pathway yields chorismic acid—a precursor in the aromatic amino acid biosynthetic pathways. This pathway portrays a prime regulatory link of primary and secondary metabolism, with Phe serving as an initiator/regulatory metabolite in the biosynthesis of phenylpropanoids of which the flavonoid pathway is a downstream branch [27,28].

Data analysis revealed a decrease in chorismic acid levels in the NS 5655 (ST) cultivar which was, however, not annotated in the NS 5511 (BT) cultivar (Table 1). As stated previously, chorismic acid provides a carbon skeleton in the synthesis of aromatic acids, from which aromatic secondary metabolites arise [27,28,51]. Benzoic acid was also detected in both cultivars. Benzoic acid and derivatives are known for antioxidant and antimicrobial activity. The decrease in levels of chorismic- and benzoic acid can be attributed to channelling of the metabolites into synthesis of various defence-related compounds to which the molecules serve as precursors [53]. 

Two major groups of phenolic compounds derived from the phenylpropanoid pathway and regarded as the major phenolic compounds found in sorghum, namely flavonoids and HCAs, accumulated significantly in infected plants. The profusion of these classes of compounds amongst the putatively identified metabolites (Figure 4A and Table 1) suggests a pivotal role in sorghum defence (as either preformed phytoanticipins or induced phytoalexins) [54,55]. Phenolic secondary metabolites are a major group directly involved in plant resistance and in determining resistance/susceptibility of a plant host to microbial pathogens [56,57]. Many reports have demonstrated accumulation of phenolic compounds at the site of infection following pathogen invasion [56,58]. 

#### 3.4.1. Flavonoids as Biomarkers in Sorghum Defence Responses 

The flavonoids annotated from extracts of infected plants (Table 1) were mostly sugar-conjugated and belonged to various subgroups; (i) flavones: apigenin and derivatives, luteolin and tricetin derivatives, (ii) flavanones: naringenin and hesperitin derivatives and (iii) flavonols: kaempferol and quercetin derivatives. Interestingly, the 3-deoxyanthocyanidins, luteolinidin and apigeninidin were not detected in any extract. 

Most of the detected flavonoid glycosides significantly accumulated following bacterial infection. Flavonoids are a highly diverse class of secondary metabolites [59], with a wide range of biological functions in plant systems which include signalling, abiotic and biotic stress response, and antioxidant activity, amongst others. Synthesis, transportation and allocation of this class of compounds hallmark an adaptive metabolism in plants geared towards protective and regulatory functions [58,59,60]. For example, upon pathogen challenge, flavonoids accumulate at the infection site and impede fungal spore germination, inactivate bacterial pathogen adhesion and distort microbial membranes, all in attempt to hinder microbial invasion [29,58]. 

**Figure 6:**
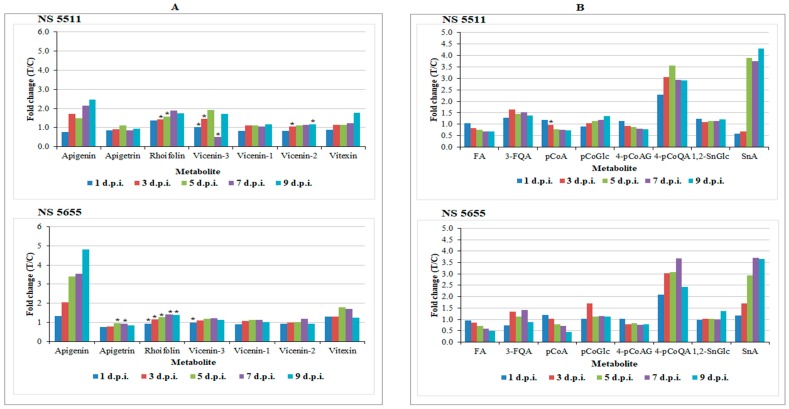
Relative quantification of some flavonoids (**A**) and hydroxycinnamic acids (**B**) annotated in sorghum leaves responding to infection by *B. andropogonis*. The relative levels of each metabolite are expressed as fold changes, computed from treated against control (T/C) samples, where fold change > 1 represents significant accumulation in NS 5511 (BT) and NS 5655 (ST). Abbreviations: (**A**) apigetrin = apigenin 7-*O*-glucoside; rhoifolin = apigenin 7-*O*-neohesperidoside; vicenin-3 = apigenin 8-C-xyloside-6-C-glucoside; vicenin-1 = apigenin 6-C-xyloside-8-C-glucoside; vicenin-2 = apigenin-6,8-di-C-glucoside; vitexin = apigenin-8-C-glucoside. (**B**) FA = ferulic acid; 3-FQA = 3-feruloylquinic acid; *p*CoA = *p*-coumaric acid; *p*CoGlc = 1-*O*-coumaroyl-beta-d-glucose; 4-*p*CoAg = 4-coumaroylagmatine; 4-*p*CoQA = 4-coumaroylquinic acid; 1,2-SnGlc =1,2-bis-*O*-sinapoyl-beta-d-glucoside; SnA = sinapoyl alcohol. The fold changes of the various treatment days presented in the graphs have a *p*-value < 0.05, except for the ones indicated by * with *p*-value > 0.05 but < 0.5.

**Figure 7:**
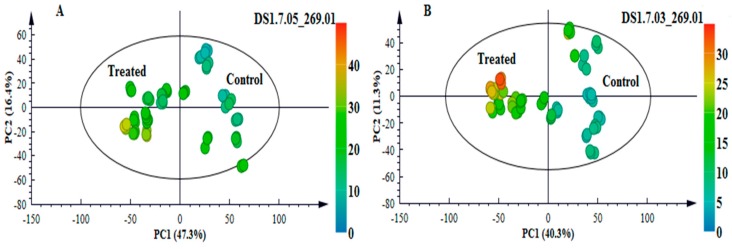
An unsupervised colour-coded PCA score plot displaying the presence/absence and intensity of the phytoalexin apigenin across the samples. (**A**): NS 5511 (BT) and (**B**): NS 5655 (ST). The absence of the metabolite in control samples and presence in samples from infected plants indicates that the metabolite was pathogen-induced.

The results revealed infection-triggered metabolic changes in flavonoid metabolism, largely characterised by significant accumulation of apigenin and its glycosides (mostly existing as *C*-glycosides) (Table 1). Apigenin demonstrated to be a pathogen-induced biomarker in both cultivars and displayed an increase in levels over time points particularly from 3–9 d.p.i. (levels ≥ 1.5-fold) (Figure 6A). This finding was visually confirmed by exploration of the PCA scores space (Figure 7). In the non-infected samples, there was no detectable presence of the metabolite. However, it was clearly detectable in the treated samples, with levels increasing over time and most intensely in NS 5655 (ST). Apigenin is a known phytoalexin in sorghum and studies have shown rapid de novo synthesis and elevated accumulation of the metabolite following infection. The flavone has been demonstrated to inhibit fungal growth and spore germination against fungal pathogens such as *Colletotrichum sublineolum* [60]. For bacterial pathogens, apigenin has been reported to exhibit a stronger antibacterial activity towards Gram-negative bacteria [61]. 

For NS 5655 (ST), levels of apigenin was slightly higher than in NS 5511 (BT). On the other hand, levels of apigenin glycosides (found to be constitutively present in this study i.e., as phytoanticipins) appeared to be more augmented in the NS 5511(BT) cultivar compared to NS 5655 cultivar (ST). The apigenin conjugates, rhoifolin and vitexin, found to significantly accumulate in infected tissues (Figure 6A), have been reported to possess antimicrobial properties [61,62]. Notably, vitexin (and related conjugates) and the other apigenin glycosides, vicenin-1, vicenin-2 and vicenin-3, were reported, in connection with defence responses. The levels of these metabolites together with those of apigetrin (Figure 6A) in infected plants suggests a defence-related role towards *B. andropogonis;* however, further work is required to elucidate and explore the particular roles in sorghum defence. 

Other flavonoids found to be up-regulated following bacterial infection (Table 1 and Figure S11) are luteolin-, naringenin-, quercetin- and chalcone conjugates. These conjugates have been linked to defence in plants following pathogen challenge. Luteolin 7-*O*-neohesperidoside has been reported as an antibacterial compound, while luteolin 7-*O*-glucoside has been shown to possess antifungal activity contributing to host resistance [61,62]. Naringenin 7-*O*-beta-d-glucoside and naringin were reported to exhibit antimicrobial activity in grains like wheat [58] and barley [63], contributing to host resistance. Similarly, antibacterial and antifungal activity has been reported for chalcones and quercetin derivatives, respectively [58,64].

**Figure 8:**
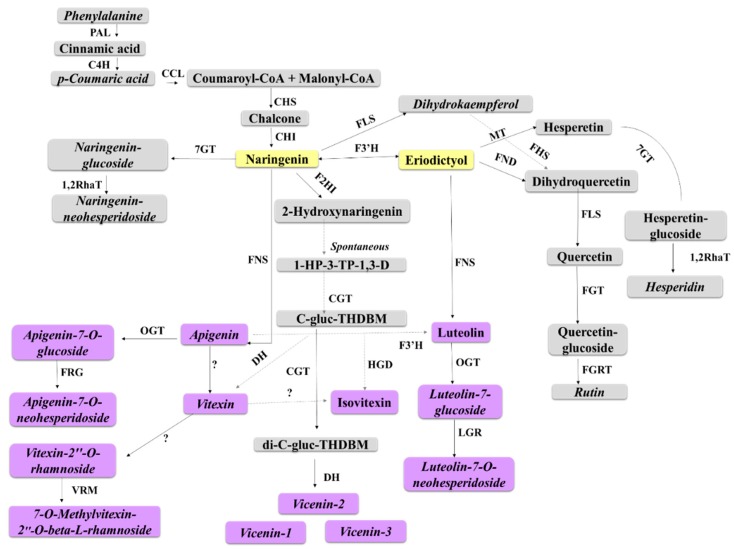
Schematic representation of the proposed biosynthetic pathway of defence-related flavonoids in sorghum. This pathway displays some of the flavonoids annotated in sorghum plant extracts following infection with *B. andropogonis*. Solid lines represent main routes and dashed lines represent alternative routes. Abbreviations: PAL = phenylalanine ammonia lyase; C4H = cinnamate 4-hydroxylase; CCL = coumaryl-CoA ligase; CHS = chalcone synthase; CHI = chalcone isomerase; 7GT = flavanones-7-*O*-glucosyltransferase; Cm1,2RhaT = 1,2 rhamnosyltransferase; F2HI = flavanone-2-hydrohlyase; FNS = flavone synthase; OGT = *O*-glycosyltransferase; FRG = flavanones 7-*O*-glucoside-2”-*O*-β-l-rhamnosyltransferase; LGR = luteolin 2-*O*-β-l-rhamnosyltransferase; VRM = vitexin 2”-*O*-rhamnoside 7-*O*-methyltransferase; MT = methyltransferase; FND = flavanones 3-dioxygenase; FLS = flavonol synthase; FGT = flavonoid 3-*O*-glucosyltransferase; FGRT = flavonol-3-*O*-glucoside l-rhamnosyltransferase; FHS = flavonoid 3’,5’-hydroxylase; CGT = *C*-glycosyl transferase; HGD = 2-hydroxynaringenin-6-*C*-glucoside dehydratase; DH = dehydrogenase; ? = enzymes not yet characterised. Metabolites annotated in this study are in ***italics***. Flavones and derivatives are indicated in purple and the flavanone precursors in yellow.

We can, therefore, propose that defence responses launched towards *B. andropogonis* infection by sorghum largely involves the subgroups of flavonoids mentioned above, particularly the flavones. A schematic representation of the metabolites annotated in this study, superimposed on the flavonoid biosynthetic pathway, is shown in Figure 8. 

#### 3.4.2. The Defensive Functions of Hydroxycinnamic Acids in Sorghum

Infected sorghum plants accumulated HCAs in response to *B. andropogonis*. This class of metabolites also originates from the early phenylpropanoid pathway [27,28] and the annotated biomarkers included coumaric-, caffeic-, sinapic- and ferulic acid conjugated to various molecules such as sugars, organic acids, alcohols, aldehydes and amines (Table 1). These compounds accumulated in varying degrees in the two cultivars. Sinapoyl alcohol, 4-coumaroylquinic acid, 3-feruloylquinic acid, 1-*O*-coumaroyl-beta-d-glucose and 1,2-bis-*O*-sinapoyl-beta-d-glucoside were up-regulated (Table 1) following bacterial infection. Furthermore, relative quantification analysis (Figure 6B) generally showed an increase in levels over time. Comparison of the two cultivars displayed a more significant accumulation in NS 5511 (BT). *p*-Coumaric acid, ferulic acid and 4-coumaroylagmatine (also shown in Figure 6B), however, displayed slight decreases in levels across time, indicative of further metabolism such as conjugation to quinic acid.

HCAs are known to possess defence-related functions. Ferulic -, caffeic -, *p*-coumaric- and sinapic acids are functional antimicrobial compounds, and precursors to the synthesis of both inducible and constitutive defence metabolites. The metabolites are also key in structural defences as monolignol precursors of lignin and by participating in cross-linking primary cell wall polysaccharides [25,56,65]. HCA amides, such as 4-coumaroylagmatine and feruloylserotonin, are also known in the context of cell wall strengthening as well as antimicrobial compounds. A study on potato showed the accumulation of these HCA amides and other HCAs; 4-coumaroylquinic acid, feruloylquinic acid, 1-*O*-sinapoyl-beta-d-glucose, 4-hydroxycoumarin and 1-*O*-feruloyl-beta-d-glucose in resistant cultivars in response to *Phytophthora infestans.* Similarly, a number of these metabolites reported in potato, and also reported here for sorghum, were associated with pathogen resistance in some members of the Poaceae family such as wheat and barley [25,26,56]. The observed significant accumulation of these HCAs can thus be linked to defence-related functions in sorghum towards *B. andropogonis*.

Sinapoyl alcohol derived from cinnamic acid via *p*-coumaric-, caffeic-, ferulic- and sinapic acid intermediates, is an important precursor (together with sinapaldehyde also annotated in this study) in plant cell wall lignification. The decrease in levels of 1-*O*-sinapoyl-beta-d-glucose (not shown) accompanied by higher levels of its active form, sinapoyl alcohol (Figure 8B), displayed a conversion of an inactive to an active form which marks the activation of structural defences to hinder pathogen penetration. 

The sinapoyl glucoside and 1-*O*-feruloyl-beta-d-glucose are inactive storage forms that are activated due to pathogen infection to form sinapoyl alcohol and feruloyl alcohol respectively [39,66]. Other metabolites annotated in this study, and also involved in structural defence, are coniferyl acetate, sinapaldehyde glucoside and sinapoyl-(S)-malate. The significant accumulation of HCA conjugates associated with a decrease in the precursors, therefore, shows channelling of the latter into synthesis of corresponding derivatives more important for sorghum resistance [26,56,67].

## 4. Materials and Methods

### 4.1. Sorghum Plant Cultivation

Two commercial cultivars (NS 5511/BT and NS 5655/ST) were evaluated and compared in this study. Both cultivars are grain sorghum hybrids (from the same breeding line) of the malting class; NS 5511 seeds (bitter) have dark testa and high condensed tannin content, while NS 5655 seeds (sweet) have light coloured testa and no condensed tannins (Agricol Seed Company, Silverton, Pretoria, RSA). NS 5511 and NS 5655 have both a rating of ‘3’ (on a 1–9 scale, 1 = most resistant and 9 = most susceptible) with regards to resistance against head smut, leaf disease and stem rot (Capstone Seeds, Howick, South Africa). Seeds were initially surface-sterilised with a 1.2% sodium hypochlorite solution for 20 min before rinsing in sterile distilled water and being placed on wet paper towel in glass Petri dishes and incubated at 27 °C in the dark for 48 h to germinate. Following germination, the seedlings were planted in vermiculite under a 12 h fluorescence light (≈ 85 µmol m^−2^ s^−2^) and 12 h dark cycle at 22–27 °C. The study was designed to monitor the responses for 9 d post-inoculation (d.p.i.); and at each time point (1, 3, 5, 7 and 9 d.p.i.) 3 biological replicates were harvested. Plants were watered twice a week with a water-soluble chemical fertiliser (Multisol ‘N’, Culterra, Muldersdrift, South Africa) and distilled water solution. All the plants were grown at the same time under the same environmental conditions to minimise biological variation. 

### 4.2. Bacterial Suspension Preparation and Infection of Sorghum Seedlings

The initial bacterial stock of *B. andropogonis* (strain BD256) was obtained from the Plant Protection Research Institute (PPRI, Agriculture Research Council, Pretoria, South Africa), cultured in Nutrient broth (Merck, Johannesburg, South Africa) and incubated overnight on a shaker at 28 °C and 130 rpm. Bacterial cells were harvested by means of centrifugation at 9 000 × *g* and 4 °C for 20 min. The pelleted cells were resuspended in 2 mL phosphate buffered saline (PBS). The working bacterial suspension was subsequently prepared by serial dilution in PBS solution to an OD_600_ of 0.1. To complete the preparation of the bacterial suspension, a wetting and spreading agent (Insure pH buffer) was added at a 1:1000 *v*/*v* dilution as per manufacturer’s (Plaaskem, Boksburg, South Africa) instructions.

At the 4-leaf growth stage (~30 d post sowing), the seedlings were infected by spraying the leaves equally and homogenously with the bacterial suspension (OD_600_ = 0.1) using a hand sprayer. In prior optimisation experiments, it was found that the spray treatment did not elicit any detectable responses in the control plants and the negative controls were accordingly not sprayed. Infected plants were then incubated at 30 °C in a high humidity environment for 24 h. Incubation was followed by exposing the plants to initial growth conditions. Following the infection, leaves from both cultivars were harvested at 1, 3, 5, 7 and 9 d.p.i. for infected plants and at 1, 5 and 9 d.p.i. for the non-infected plant leaves. Leaves were cut from the plant and immediately stored at −80 °C to quench enzymatic activity until the metabolite extraction steps could be performed.

### 4.3. Metabolite Extraction and Pre-Analytical Sample Preparation

Metabolites were extracted as previously described [20]. Briefly, leaves from infected and non-infected plants were mixed with a cold extraction solvent (80% aqueous methanol) in a ratio of 1:15 (*w*/*v*). The mixture was homogenised using an Ultra-Turrax homogeniser, followed by sonication for 15 s with a probe sonicator (Bandelin Sonopuls, Berlin, Germany) set at 55% power. Homogenates were centrifuged at 5000× *g* and 4 °C for 25 min. The supernatants of each sample were concentrated by evaporation under vacuum to 1 mL using a rotary evaporator set at 55 °C. The 1 mL extracts from each sample were further evaporated to complete dryness with a speed vacuum concentrator (Eppendorf, Merck, Johannesburg, South Africa) set at 45 °C. The final step of sample preparation consisted of resuspending the dried extracts in 50% UHPLC-grade methanol (Romil, Cambridge, UK) in a 1:10 *m*/*w* ratio. This was followed by filtering samples through 0.22 µm nylon syringe filters into UHPLC glass vials fitted with 500 µL inserts. The filtered samples were capped and kept at −20 °C until analysed. 

### 4.4. Ultra-High Performance Liquid Chromatography-High Definition Mass Spectrometry 

UHPLC-MS analyses were performed on a Waters Acquity UHPLC coupled in tandem to a SYNAPT G1 Q-TOF mass spectrometer (Waters Corporation, Milford, MA, USA) via an electrospray ionisation (ESI) interface. Sample extracts were chromatographically separated on a reverse phase C18 column (150 mm × 2.1 mm × 1.8 µm—HSS T3, Waters Corporation, Milford, MA, USA) at 60 °C. The mobile phase consisted of 0.1% formic acid in MilliQ water (solvent A) and 0.1% formic acid in acetonitrile (Romil, Cambridge, UK) (solvent B), and flow rate was set to 0.4 mL/min. Gradient elution was used and the initial conditions were 2% B and maintained for 1 min. The gradient was ramped to 95% B at 15 min and maintained for 2 min, and then changed to the initial conditions at 18 min, followed by a 2 min equilibration time of the column. The total chromatographic run time was 20 min and the injection volume was 2 µL. Each sample was analysed in triplicate. Sample acquisition was randomised and the QC sample used to condition the UHPLC-MS system was repeatedly injected to account for any analytical variability. The MS detector acquired data in both positive and negative modes following electrospray ionisation (ESI). The conditions were set as follows: capillary voltage of 2.5 kV, sampling cone at 30 V, extraction cone at 4 V, cone gas flow 50 L h^−1^, desolvation gas flow 550 L h^−1^, source temperature at 120 °C, desolvation temperature at 450 °C, scan time of 0.1 s and mass range of 100–1000 Da. Leucine encephalin (50 pg/mL) was used as a calibrant to acquire mass accuracies between 1 and 3 mDa and data were acquired at different collision energies (MS^E^, 10–50 eV) to aid with structural elucidation and annotation of the analytes. 

### 4.5. Data Processing and Multivariate Data Analyses 

Raw data, both ESI negative and positive, obtained from UHPLC-HDMS were extracted using MassLynx^TM^ XS software and processed with MarkerLynx software (Waters Corporation, Manchester, UK). The data matrices obtained from MarkerLynx processing were exported into SIMCA 14 (Umetrics, Umea, Sweden) for multivariate statistical analyses. The data were Pareto-scaled before principal component analysis (PCA) and orthogonal projection to latent structures discriminant analysis (OPLS-DA). For the purposes of the MVDA, the data sets from all the control time points were combined and compared to the combined data sets obtained from the infected tissues at different days. The generated models were validated using different methods. The calculated OPLS-DA models were statistically significant models (with CV-ANOVA *p*-value less than 0.05), and computed to separate multivariate relationships into: predictive variation (related to bacterial infection) and orthogonal variation (unrelated to bacterial infection) [8,9,10,11,12]. Features significantly contributing to the model, with │p[1]│ ≥ 0.05 and │p(corr)│ ≥ 0.5, were extracted from the OPLS-DA loading S-plot for downstream metabolite annotation. Fold changes indicated in Table 1 were obtained from the OPLS-DA model of all control samples (days 1, 5, 9) computed against all samples from infected tissues (days 1, 3, 5, 7, 9), whilst the fold change values used to compute the graphs in Figure 5 and Figure 6 were dependant on the model of a control (C 1 day) vs. samples from a particular day post infection.

### 4.6. Metabolite Annotation

Metabolites were annotated as previously described [9,20]. To summarise, metabolite annotation involved running data matrices obtained after MarkerLynx processing (raw data) on the Taverna workbench (www.taverna.org.uk) for PUTMEDID_LCMS metabolite identification workflows. The workflows consist of correlation analysis, metabolic feature annotation and metabolite annotation. The resulting metabolite identities generated were then confirmed with the aid of MS fragmentation patterns acquired at different collision energies (MS^E^). Here, accurate masses obtained from the SYNAPT G1 Q-TOF system were used to generate empirical formulae. Corresponding formulae with mass difference between measured and calculated mass at or below 5 mDa, was selected and queried against various available online databases. Parameters such as isotopic fit (iFit) and double bond equivalent (DBE) were taken into consideration in the selection of the formulae. Plant metabolite databases that included the Dictionary of Natural Products [68] and the Plant Metabolic Network [69] were used to aid in metabolite annotation. Literature available on metabolites from sorghum (related species as well as other plant species) was also used for comparative assessment and metabolite identity confirmation [55,70,71,72,73,74,75,76]. Thus, annotation was done at level 2 of the Metabolomics Standards Initiative (MSI) [77]. The diagnostic fragments (obtained at various energy levels) which aided metabolite annotation for some of the metabolites are shown in Table S2.

## 5. Conclusions

The dynamic changes and relative quantitative analyses observed in metabolite levels of the NS 5511 and NS 5655 cultivars of *Sorghum bicolor* explain the differences visualised in LC-MS chromatograms as well as the clustering patterns of samples in the PCA scores plots, i.e., time-related differences reflecting changing metabolite profiles. Moreover, the presence/absence of metabolites and varying degrees in metabolite accumulation in the two cultivars point to underlying differential metabolism between the two. These results show that both cultivars respond to pathogen attack, but that the ‘defensomes’ to *B. andropogonis* diverge. This was found to be due to differential metabolic reprogramming that contributes to and explains the variances in the phenotypic differences in the degrees of disease tolerance. The observed nuances can be attributed to genetic factors and aspects controlling the kinetics of the induced defence responses, and the extent to which activation occurs [20,78]. 

Based on the disease severity-rating index, the NS 5511 (BT) cultivar demonstrated to be more tolerant compared to the NS 5655 (ST) cultivar, as it symptomatically displayed delayed signs of wilting and spreading of lesions compared to the latter. This observation corroborates with Tugizimana et al., [20], and is supported by the early accumulation (1–5 d.p.i.) of the phytohormone SA to significant levels in the NS 5511 cultivar—important in initiating and orchestrating defence responses and systemic resistance. In the NS 5655 cultivar, the levels of the hormone appeared to increase only at later stages of the treatment. The above observation suggests that timing and intensity of the accumulation of crucial defence metabolites is essential to mount an effective resistant state. Earlier accumulation and increased levels greatly potentiates the launching of prompt and effective defence responses i.e., in conferring the resistance phenotype, and in the event of the opposite, the efficiency of immune responses may be lessened [34,79]. Similarly, the accumulation of the apigenin glycosides, more significantly in the NS 5511 cultivar, probably contributed to disease tolerance/resistance. The presence of defence-related metabolites (phytoanticipins) in plants creates a condition of ‘readiness’ that, in the event of pathogen attack, the metabolites act as a first line of chemical defence to inhibit pathogen proliferation. Additionally, hydrolysis of preformed conjugated phytoanticipins to rapidly generate phytoalexins [65] aimed to further limit pathogen proliferation, and also contributes to plant resistance [79]. Therefore, a rapid increase of antimicrobial metabolite levels significantly contributes to the plant resistance phenotype [22,79]. Noteworthy, however, is that the functional roles of phytoanticipins and phytoalexins are somewhat overlapping and can be difficult to clearly distinguish at certain stages of the infection. In summary, the picture depicted by the results from this untargeted metabolomics study demonstrates metabolic reprogramming of both primary and secondary pathways in *S. bicolor* following *B. andropogonis* infection, and the spanning of an array of defence-related metabolites aimed at establishing an enhanced defensive capacity. Furthermore, the results reveal that the phenylpropanoid and flavonoid metabolic pathways are central in *S. bicolor* defence against to *B. andropogonis*. 

## Figures and Tables

**Table 1:** Annotation of discriminatory metabolites belonging to various chemical classes and related to *Burkholderia andropogonis*-induced metabolic reprogramming in *Sorghum bicolor* plants. Following UHPLC-MS analysis, the selected features from both ESI (+/−) modes, were extracted from OPLS-DA S-plots and annotated at MSI-level 2. The reported fold changes for cultivars NS 5511 and NS 5655 were obtained from an OPLS-DA model of control vs. all treated/infected samples (1–9 d.p.i.).

Metabolites	*m/z*	RT (min)	Adduct	Ion Mode	Molecular Formula	NS 5511		NS 5655		Metabolite Class
						*p*-Value	Fold Change	*p*-Value	Fold Change	
l-Phenylalanine	180.092	2.59	[M−H_NH_3_]^−^	neg	C_9_H_11_NO_2_	0.159	0.9	0.002	0.9	Amino acid
l-Tyrosine	182.081	1.13	[M+H]^+^	pos	C_9_H_11_NO_3_	1.77 × 10^−31^	0.5	1.94 × 10^−31^	0.4	Amino acid
l-Tryptophan	205.097	2.69	[M+H]^+^	pos	C_11_H_12_N_2_O_2_	1.87 × 10^−27^	0.6	2.67 × 10^−15^	0.5	Amino acid
Benzoic acid	121.028	4.46	[M−H]^−^	neg	C_7_H_6_O_2_	1.76 × 10^-20^	0.6	0.603	0.7	Benzoates
Dhurrin	334.090	2.6	[M+H_Na]^+^	pos	C_14_H_17_NO_7_	2.49 × 10^−29^	0.4	2.51 × 10^−27^	0.4	Cyanogen
Apigenin	269.007	7.05	[M−H]^−^	neg	C_15_H_10_O_5_	0.838	1.0	0.000	1.5	Flavonoid
Protocatechuic acid hexoside	315.069	5.41	[M−H]^−^	neg	C_13_H_16_O_9_	5.24 × 10^−7^	1.5	1.67 × 10^−11^	2.1	Flavonoid
Tricin	329.066	8.92	[M−H]^−^	neg	C_17_H_14_O_7_	6.62 × 10^−7^	0.7	0.091	0.8	Flavonoid
Sophoraflavanone B	341.137	2.50	[M+H]^+^	pos	C_20_H_20_O_5_	8.06 × 10^−34^	0.4	4.38 × 10^−27^	0.4	Flavonoid
Trihydroxypentamethoxyflavone	419.095	5.69	[M−H]^−^	neg	C_20_H_20_O_10_	1.64 × 10^−24^	3.1	1.31 × 10^−16^	3.8	Flavonoid
Sophoraflavanone G	423.182	5.46	[M−H]^−^	neg	C_25_H_28_O_6_	4.59 × 10^−11^	0.6	1.29 × 10^−5^	0.7	Flavonoid
Apigenin-8-*C*-glucoside (vitexin)	431.099	5.55	[M−H]^−^	neg	C_21_H_20_O_10_	7.57 × 10^−5^	1.4	0.347	1.1	Flavonoid
Apigetrin (apigenin 7-*O*-glucoside)	431.098	6.33	[M−H]^−^	neg	C_21_H_20_O_10_	0.238	1.0	0.001	0.8	Flavonoid
Naringenin 7-*O*-beta-d-glucoside (prunin)	433.114	5.91	[M−H]^−^	neg	C_21_H_22_O_10_	1.44 × 10^−16^	2.3	5.76 × 10^−18^	4.6	Flavonoid
Luteolin 7-*O*-glucoside	447.091	5.71	[M−H]^−^	neg	C_21_H_20_O_11_	4.44 × 10^−13^	1.6	0.799	1.0	Flavonoid
Quercetin 3-*O*-rhamnoside (quercitrin)	447.092	4.61	[M−H]^−^	neg	C_21_H_20_O_11_	9.02 × 10^−17^	1.8	8.37 × 10^−17^	3.8	Flavonoid
Pentahydroxychalcone 4′-*O*-glucoside	449.108	4.57	[M−H]^−^	neg	C_21_H_22_O_11_	7.50 × 10^−11^	1.4	5.49 × 10^−19^	2.1	Flavonoid
Apigenin 8-C-xyloside-6-C-glucoside (vicenin-3)	563.139	5.09	[M−H]^−^	neg	C_26_H_28_O_14_	0.672	1.1	3.88 × 10^−8^	1.2	Flavonoid
Apigenin 6-C-xyloside-8-C-glucoside (vicenin-1)	563.140	4.87	[M−H]^−^	neg	C_26_H_28_O_14_	6.91 × 10^−11^	1.2	0.008	1.1	Flavonoid
Vitexin 2″-*O*-rhamnoside	577.154	5.32	[M−H]^−^	neg	C_27_H_30_O_14_	1.43 × 10^−15^	1.5	7.43 × 10^−14^	1.5	Flavonoid
Apigenin 7-*O*-neohesperidoside (rhoifolin)	577.156	6.06	[M−H]^−^	neg	C_27_H_30_O_14_	1.52 × 10^−7^	1.1	0.488	1.0	Flavonoid
Unknown flavonoid	581.149	4.33	[M−H]^−^	neg	C_26_H_30_O_15_	7.67 × 10^−25^	2.5	1.75 × 10^−15^	2.8	Flavonoid
Luteolin 7-*O*-neohesperidoside	593.150	5.51	[M−H]^−^	neg	C_27_H_30_O_15_	2.20 × 10^−15^	1.3	0.037	0.9	Flavonoid
Apigenin-6,8-di-C-glucoside (vicenin-2)	593.151	4.45	[M−H]^−^	neg	C_27_H_30_O_15_	0.637	1.0	8.60 × 10^−6^	1.3	Flavonoid
Quercetin-3-rhamnoside-7-rhamnoside	595.165	4.51	[M−H]^−^	neg	C_27_H_32_O_15_	2.87 × 10^−9^	1.4	4.17 × 10^−10^	1.5	Flavonoid
Quercetin rutinoside (rutin)	609.146	5.43	[M−H]^−^	neg	C_27_H_30_O_16_	1.22 × 10^−19^	2.1	3.98 × 10^−10^	2.1	Flavonoid
Hesperidin	609.181	4.80	[M−H]^−^	neg	C_28_H_34_O_15_	0.236	1.1	0.000	0.9	Flavonoid
Unknown flavonoid	611.158	3.10	[M−H]^−^	neg	C_27_H_32_O_16_	4.59 × 10^−9^	1.2	3.07 × 10^−8^	1.4	Flavonoid
Naringenin 7-*O*-neohesperidoside (naringin)	625.180	3.33	[M−H_HCOOH]^−^	neg	C_27_H_32_O_14_	0.000	1.1	3.10 × 10^−6^	0.7	Flavonoid
7-*O*-Methylvitexin 2″-*O*-beta-l-rhamnoside	637.177	6.21	[M−H_HCOOH]^−^	neg	C_28_H_31_O_14_	0.466	1.0	0.563	1.0	Flavonoid
4-Hydroxycoumarin	161.024	1.87	[M−H]^−^	neg	C_9_H_6_O_3_	3.91 × 10^−12^	0.6	0.017	0.8	HCA
*p*-Coumaric acid	163.039	3.65	[M−H]^−^	neg	C_9_H8O_3_	0.064	0.9	0.953	0.9	HCA
Caffeic acid	179.034	4.35	[M−H]^−^	neg	C_9_H_8_O_4_	1.68 × 10^−7^	0.5	0.083	0.6	HCA
Ferulic acid	193.048	4.01	[M−H]^−^	neg	C_10_H_10_O_4_	1.95 × 10^−12^	0.8	1.25 × 10^−7^	0.8	HCA
Sinapoyl alcohol	209.074	6.72	[M−H]^−^	neg	C_11_H_14_O_4_	1.48 × 10^−15^	2.4	1.07 × 10^−16^	3.1	HCA
Coniferyl acetate	221.081	7.42	[M−H]^−^	neg	C_12_H_14_O_4_	nd	nd	3.47 × 10^−10^	8.2	HCA
2-*O*-Caffeoylglyceric acid	267.048	4.38	[M−H]^−^	neg	C_12_H_12_O_7_	1.48 × 10^−10^	0.5	0.017	0.7	HCA
4-Coumaroylquic acid	337.051	3.29	[M−H]^−^	neg	C_16_H_18_O_8_	1.54 × 10^−30^	3.8	6.41 × 10^−26^	3.6	HCA
Caffeic acid hexose	341.083	6.17	[M−H]^−^	neg	C_15_H_18_O_9_	3.44 × 10^−7^	0.7	0.003	0.8	HCA
4-Caffeoylquinic acid	353.091	3.58	[M−H]^−^	neg	C_16_H_18_O_9_	4.75 × 10^−23^	0.3	2.60 × 10^−5^	0.5	HCA
1-*O*-Feruloyl-beta-d-glucose	355.102	4.06	[M−H]^−^	neg	C_16_H_20_O_9_	3.21 × 10^−24^	0.2	4.17 × 10^−18^	0.1	HCA
4-Coumaroylagmatine	277.027	2.63	[M−H]^−^	neg	C_14_H_20_N_4_O_2_	0.0464	0.9	1.09 × 10^−7^	0.8	HCA
3-Feruloylquinic acid	367.099	3.75	[M−H]^−^	neg	C_17_H_20_O_9_	5.98 × 10^−9^	1.3	0.002	1.2	HCA
Sinapoyl aldehyde	371.130	6.53	[M+H]^+^	pos	C_17_H_22_O_9_	4.70 × 10^−29^	0.3	3.58 × 10^−22^	0.4	HCA
2-*O*-Caffeoylglucarate	371.062	2.05	[M−H]^−^	neg	C_15_H_16_O_11_	0.167	0.3	nd	nd	HCA
1-*O*-Coumaroyl-beta-d-glucose	371.097	4.26	[M−H_HCOOH]^−^	neg	C_15_H_18_O_8_	0.001	1.1	0.442	1.0	HCA
Sinapoyl-(S)-malate	385.078	3.74	[M−H_HCOOH]^−^	neg	C_15_H_16_O_9_	1.56 × 10^−10^	0.8	0.066	0.6	HCA
1-*O*-Sinapoyl-beta-d-glucose	385.113	5.16	[M−H]^−^	neg	C_17_H_22_O_10_	7.38 × 10^−9^	0.8	0.000	0.9	HCA
Feruloylserotonin	351.072	3.65	[M−H]^−^	neg	C_20_H_20_N_2_O_4_	0.793	1.0	1.43 × 10^−10^	0.3	HCA
1,3-*O*-Coumaroyl-feruloylglycerol	413.121	9.03	[M−H]^−^	neg	C_22_H_22_O_8_	9.84 × 10^−12^	0.4	4.47 × 10^−17^	0.5	HCA
Sinapaldehyde glucoside	415.123	4.44	[M−H_HCOOH]^−^	neg	C_17_H_22_O_9_	7.13 × 10^−16^	0.7	9.48 × 10^−7^	0.8	HCA
1,3-*O*-Diferuloylglycerol	443.132	9.22	[M−H]^−^	neg	C_23_H_24_O_9_	4.20 × 10^−11^	0.4	2.53 × 10^−26^	0.3	HCA
Caffeic acid derivative	475.143	1.92	[M−H]^−^	neg	C_20_M_28_O_13_	1.81 × 10^−15^	1.5	2.56 × 10^−17^	2.5	HCA
1,2-bis-*O*-Sinapoyl-beta-d-glucoside	591.166	6.19	[M−H]^−^	neg	C_28_H_32_O_14_	0.547	1.0	0.105	1.0	HCA
Indole-3-acrylic acid/*N*-Ac-indole-3-carboxyaldehyde	188.076	2.71	[M+H]^+^	pos	C_11_H_9_NO_2_	2.12 × 10^−27^	0.5	1.85 × 10^−18^	0.5	Indole
Methyl indole-3-acetate	190.085	2.69	[M+H]^+^	pos	C_11_H_11_NO_2_	4.47 × 10^−11^	0.5	4.33 × 10^−9^	0.5	Indole
Indole-3-pyruvate	202.051	7.89	[M−H]^−^	neg	C_11_H_9_NO_3_	3.07 × 10^−18^	1.9	5.79 × 10^−13^	2.5	Indole
6-Hydroxy-indole-3-acetyl-valine	289.119	3.95	[M−H]^−^	neg	C_15_H_18_N_2_O_4_	1.83 × 10^−9^	1.7	0.469	0.9	Indole
Indole-3-acetyl-leucine	333.120	3.25	[M+H_NaNa]^+^	pos	C_16_H_20_N_2_O_3_	1.45 × 10^−7^	4.1	0.001	1.9	Indole
Indole-3-yl-acetyl-myo-inositol l-arabinoside	468.152	3.13	[M−H]^−^	neg	C_21_H_27_NO_11_	0.000	1.3	0.117	0.9	Indole
DIMBOA-Glc	372.093	1.58	[M−H]^−^	neg	C_15_H_19_NO_10_	0.001	0.8	9.77 × 10^−14^	0.4	Benzoxazine
Isocitric acid	191.018	1.10	[M−H]^−^	neg	C_6_H_8_O_7_	5.97 × 10^−14^	2.3	1.64 × 10^−9^	1.6	Carboxylic acid
Octadecatetraenoic acid	275.200	13.44	[M−H]^−^	neg	C_18_H_28_O_2_	1.64 × 10^−17^	4.1	1.20 × 10^−11^	3.6	Fatty acid
16-Hydroxypalmitate	271.044	13.43	[M−H]^−^	neg	C_16_H_31_O_3_	8.38 × 10^−19^	4.9	9.55 × 10^−13^	4.1	Fatty acid
15-Hydroxylinoleic acid	295.226	14.3	[M−H]^−^	neg	C_18_H_32_O_3_	1.38 × 10^−18^	3.6	8.76 × 10^−14^	3.4	Fatty acid
10,16-Dihydroxypalmitate	287.075	10.24	[M−H]^−^	neg	C_16_H_31_O_4_	9.76 × 10^−14^	8.0	6.75 × 10^−11^	8.1	Fatty acid
Dihydroxy-octadecadienoic acid	311.220	11.81	[M−H]^−^	neg	C_18_H_32_O_4_	5.87 × 10^−17^	4.9	2.18 × 10^−11^	6.0	Fatty acid
11,12,13-Trihydroxy-9,15-octadecadienoic acid	327.215	9.06	[M−H]^−^	neg	C_18_H_32_O_5_	1.95 × 10^−32^	2.7	1.30 × 10^−5^	1.5	Fatty acid
9,12,13-Trihydroxy-10-octadecenoic acid	329.229	9.60	[M−H]^−^	neg	C_18_H_34_O_5_	3.23 × 10^−27^	2.5	3.95 × 10^−17^	2.0	Fatty acid
Salicylic acid	137.031	3.69	[M−H]^−^	neg	C_7_H_6_O_3_	4.29 × 10^−12^	1.3	0.091	3.9	Phytohormone
Salicylic acid 2-*O*-beta-d-glucoside	299.074	1.62	[M−H]^−^	neg	C_13_H_16_O_8_	7.21 × 10^−17^	1.7	9.28 × 10^−24^	1.9	Phytohormone
Jasmonoyl-l-isoleucine	322.010	4.16	[M−H]^−^	neg	C_18_H_29_NO_4_	2.94 × 10^−12^	0.5	5.03 × 10^−20^	0.1	Phytohormone
Dihydrozeatin-9-*N*-glucoside-*O*-glucoside	544.208	2.80	[M−H]^−^	neg	C_22_H_35_N_5_O_11_	0.001	0.8	4.22 × 10^−12^	2.8	Phytohormone
Zeatin riboside	352.183	3.16	[M+H]^+^	pos	C_15_H_21_N_5_O_5_	1.35 × 10^−28^	0.3	3.27 × 10^−22^	0.4	Phytohormone
Chorismic acid	225.040	2.61	[M−H]^−^	neg	C_10_H_10_O_6_	nd	nd	0.076	0.9	Shikimate
Caffeoylshikimic acid	335.076	4.64	[M−H]^−^	neg	C_16_H_16_O_8_	2.89 × 10^−17^	0.3	3.84 × 10^−11^	0.3	Shikimate/HCA

nd: not detected in the cultivar; *p*-values refer to significance level of metabolites. Fold change was calculated by dividing the average of the metabolite peak intensity values in replicate samples of ‘infected’ by the average of the metabolite intensity values in replicate samples of ‘control’, a value >1 represents an increase (metabolite is higher in the infected samples than in the control) and value <1 represents a decrease (metabolite is higher in the control and infection led to decrease in levels). HCA: hydroxycinnamic acid.

## Data Availability

The study design information, LC-MS raw data, analyses and data processing information, and the meta-data are being deposited to the EMBL-EBI metabolomics repository—MetaboLights50, with the identifier MTBLSxyz (http://www.ebi.ac.uk/metabolights/MTBLS817).

## Supplementary Materials

The following are available online at http://www.mdpi.com/2218-1989/9/1/8/s1, Table S1: Bacterial leaf stripe disease severity-rating in NS 5511 (BT) and NS 5655 (ST) sorghum cultivars. Table S2: Indicating diagnostic fragments for some of the metabolites annotated in Table 1, Figure S1: Symptom progression on sorghum leaves subsequent to infection with *B. andropogonis*, A: NS 5511 cv and B: NS 5655 cv, Figure S2: UHPLC-MS BPI chromatograms data of extracts derived from sorghum NS 5511 (BT) cultivar responding to *B. andropogonis* infection. Figure S3: UHPLC-MS BPI chromatograms of extracts derived from sorghum NS 5655 (ST) cultivar responding to *B. andropogonis* infection. Figure S4: Comparative UHPLC-MS BPI chromatograms of extracts derived from sorghum NS 5511 (BT) *vs* NS 5655 (ST) cultivars responding to *B. andropogonis*. Figure S5: PC analyses of the ESI positive data for sorghum NS 5511 (BT) extracts. Figure S6: PC analyses of the (I) ESI negative and (II) ESI positive data for sorghum NS 5655 (ST) extracts. Figure S7: PC analyses of the ESI positive data for sorghum NS 5511 (BT) and NS 5655 (ST) extracts. Figure S8: Supervised multivariate analyses of the ESI negative UHPLC-MS data for NS 5655 (ST) cultivar extracts. Figure S9: Supervised multivariate analyses of the ESI negative UHPLC-MS data for NS 5511 (BT) cultivar extracts. Figure S10: Supervised multivariate analyses of the ESI negative UHPLC-MS data for NS 5655 (ST) cultivar extracts. Figure S11: Relative quantification of flavanones and flavonols annotated in sorghum leaves responding to *B. andropogonis* infection.
