# Supplementary material for: Untargeted Metabolomics Reveal Defensome-Related Metabolic Reprogramming in Sorghum bicolor against Infection by Burkholderia andropogonis
Source: Metabolites. 2019 Jan 2;9(1):8. doi: 10.3390/metabo9010008 (PMC6359421; doi:10.3390/metabo9010008)
Supplement: Supplementary file 1 [file metabolites-09-00008-s001.pdf]

Supplementary Data File

Untargeted metabolomics reveal defensome-related metabolic reprogramming in *Sorghum bicolor* against infection by *Burkholderia andropogonis.*

Charity R. Mareya, Fidele Tugizimana, Lizelle A. Piater, Ntakadzeni E. Madala, Paul A. Steenkamp and Ian A. Dubery*

Centre for Plant Metabolomics Research, Department of Biochemistry, University of Johannesburg, Auckland Park, 2006, Johannesburg, South Africa
* idubery@uj.ac.za; Tel.: +27-011-559-2401; Fax: +27-011-559-2370.

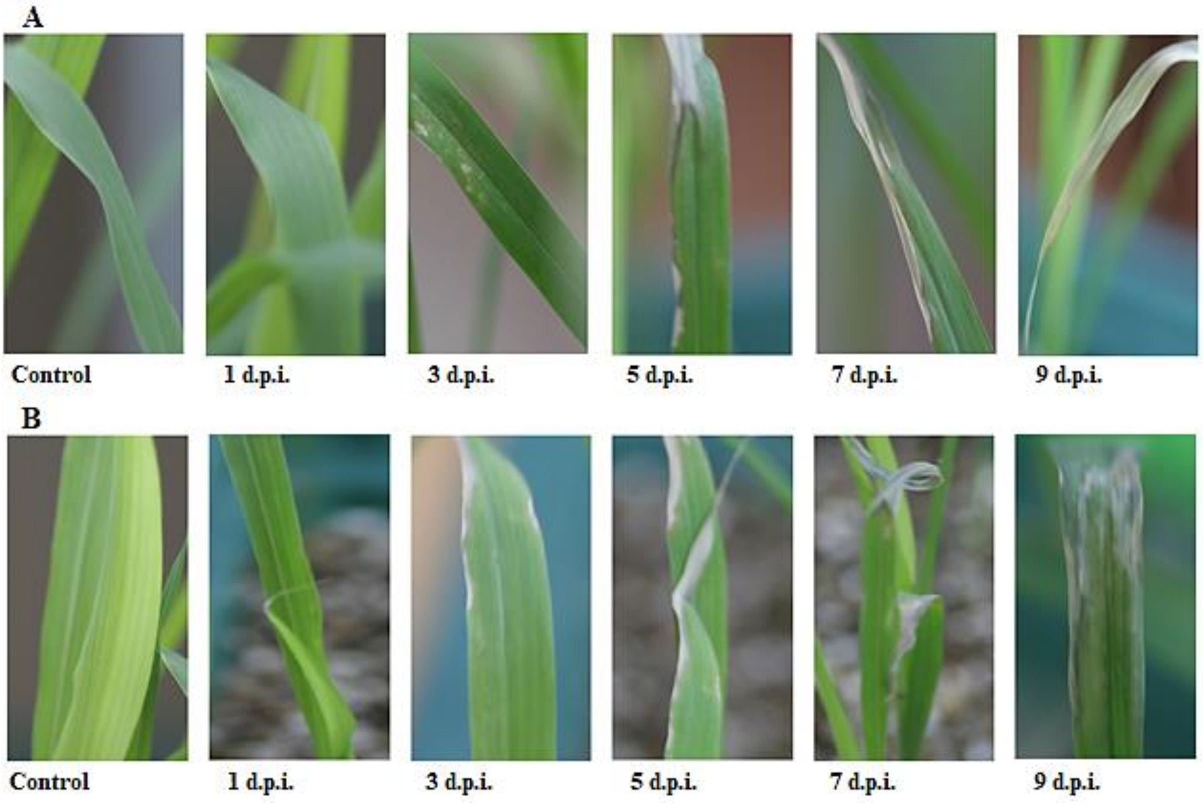

Figure S1: Symptom progression on sorghum leaves subsequent to infection with *B. andropogonis*, A: NS 5511 cv and B: NS 5655 cv. The symptoms are compared to the non-infected plants (controls) at day 1 . With the infected plants, small tan lesions started to show at 3 d.p.i. and progressed (lesion elongation) over the days to a point where most parts of the leaves were covered in lesions and drying of leaves could be noticed (9 d.p.i.).

Table S1: Bacterial leaf stripe disease severity-rating in NS 5511 (BT) and NS 5655 (ST) sorghum cvs.

Disease severity index according to cultivar		
**Days post inoculation (d.p.i.)**	**NS 5511 (BT)**	**NS 5655 (ST)**
**1**	1	1
**2**	1	1
**3**	2	2
**4**	2	3
**5**	3	3
**6**	3	4
**7**	4	4-5
**8**	4	5
**9**	5	6

*Severity ratings index was defined as follows:

1 = no lesions.

2 = 1 - 10% lesions.

3 = 11 -25% lesions.

4 = 26 -50% lesions and signs of wilting.

5 = 51 - 75% lesions and ≤50% wilting.

A

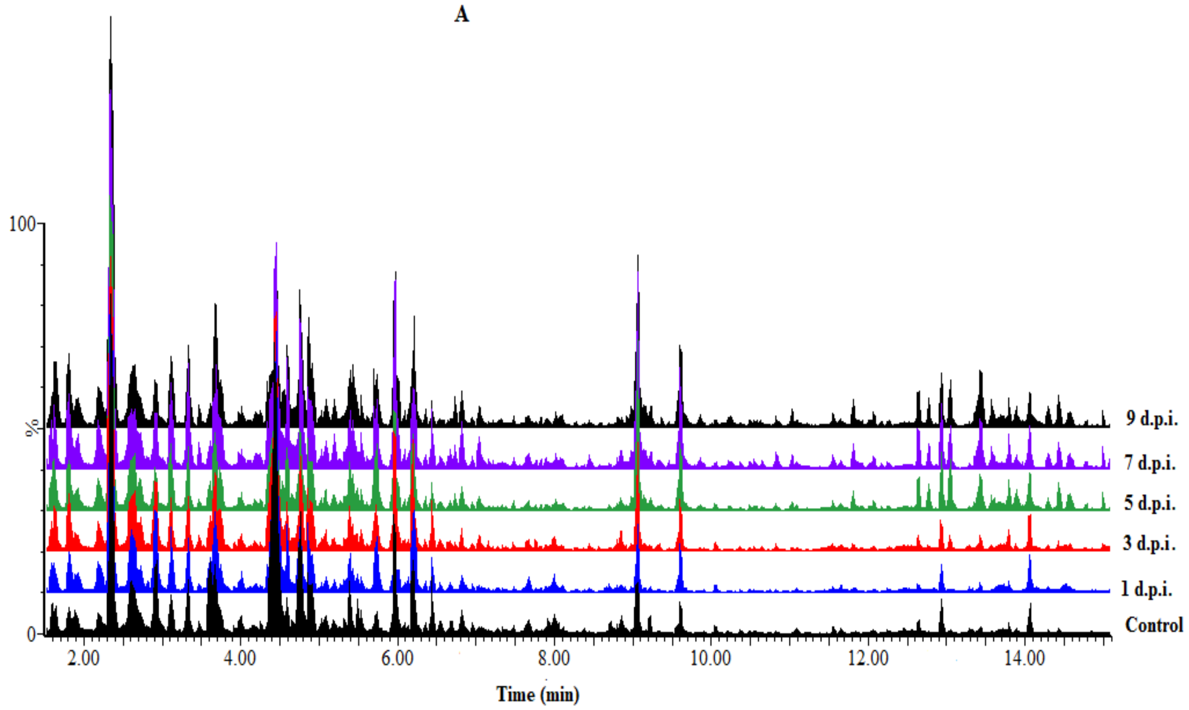

B

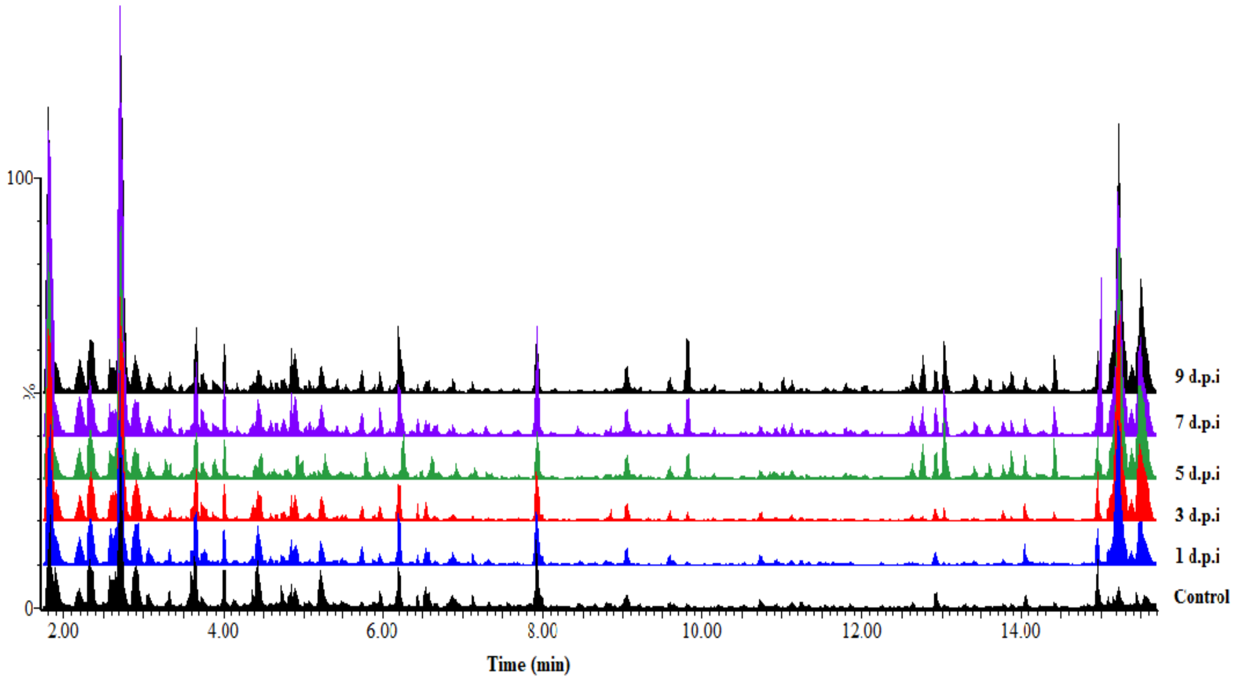

Figure S2: UHPLC-MS BPI chromatograms data of extracts derived from sorghum NS 5511 (BT) cv responding to *B. andropogonis* infection. A: ESI negative and **B: ESI positive**. Sample extracts were prepared from plant material harvested at the indicated time intervals. Variation in the displayed chromatograms, linked to treatment- and time-related metabolic changes, can be visually observed from control (non-infected) *vs* infected samples (1 - 9 d.p.i.) chromatograms.

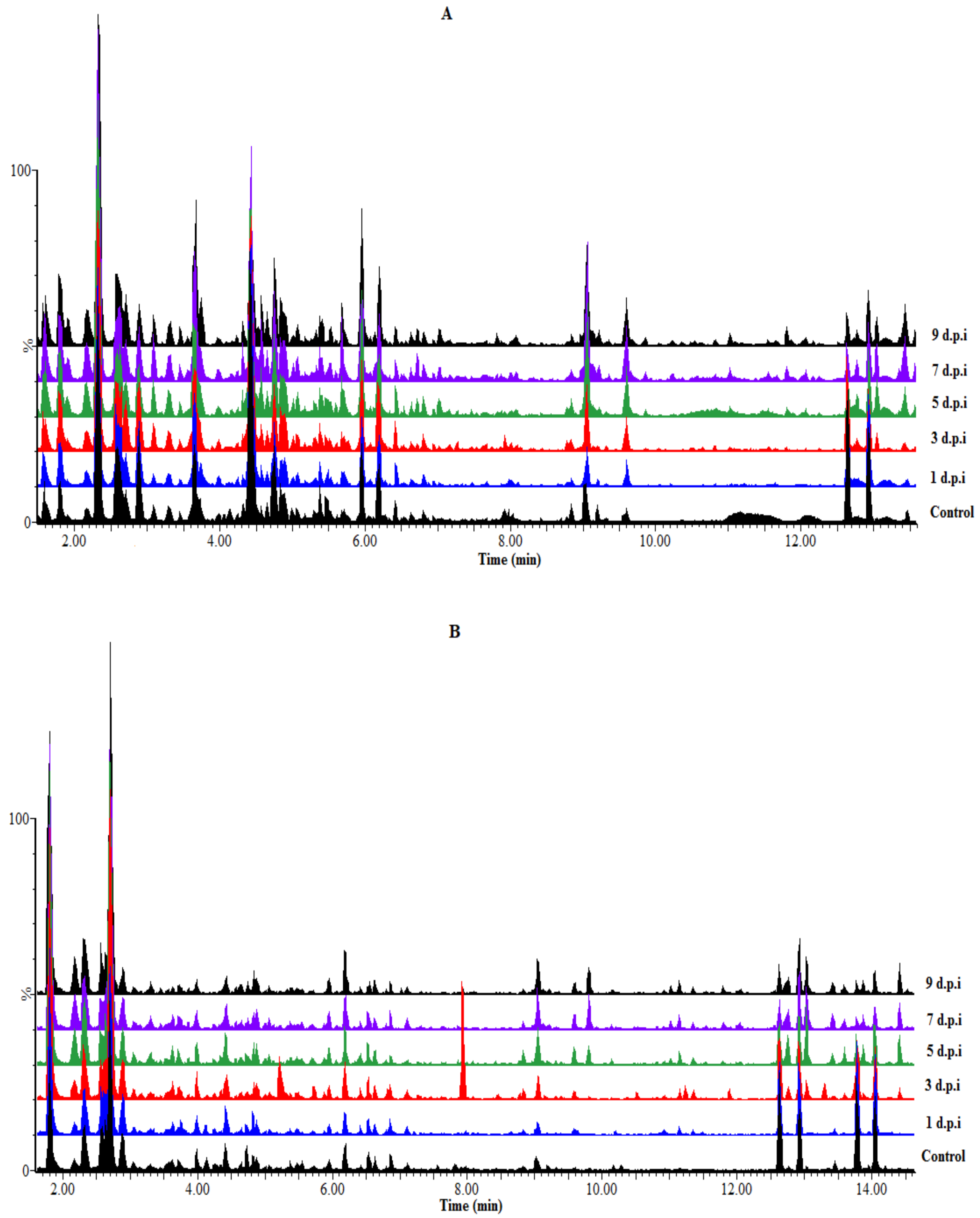

Figure S3: UHPLC-MS BPI chromatograms of extracts derived from sorghum NS 5655 (ST) cv responding to *B. andropogonis* infection. A: ESI negative and **B: ESI positive.** Control (non-treated) sample extract chromatograms are shown against chromatograms of infected sample (1 - 9 d.p.i.) extracts. The sample extracts contained a mixture of polar, mid-polar and non-polar compounds, with most being mid-polar. The chromatograms also show visually noticeable differences between the control and treated samples as well as time-dependent metabolic changes.

A

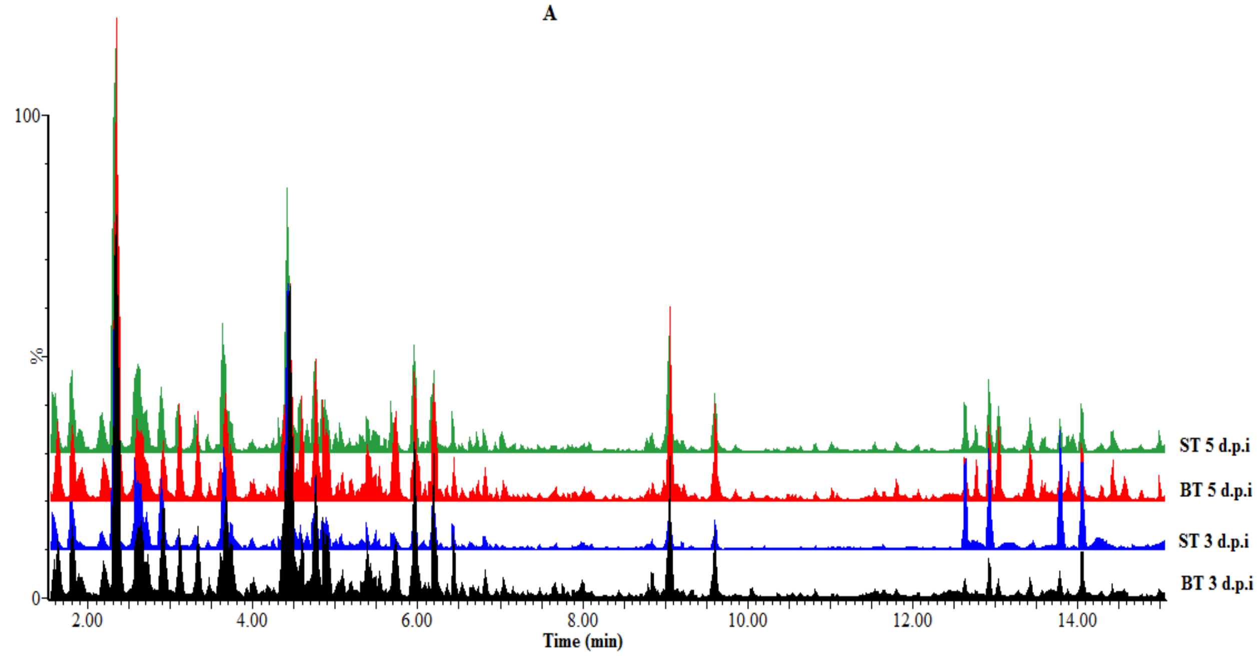

B

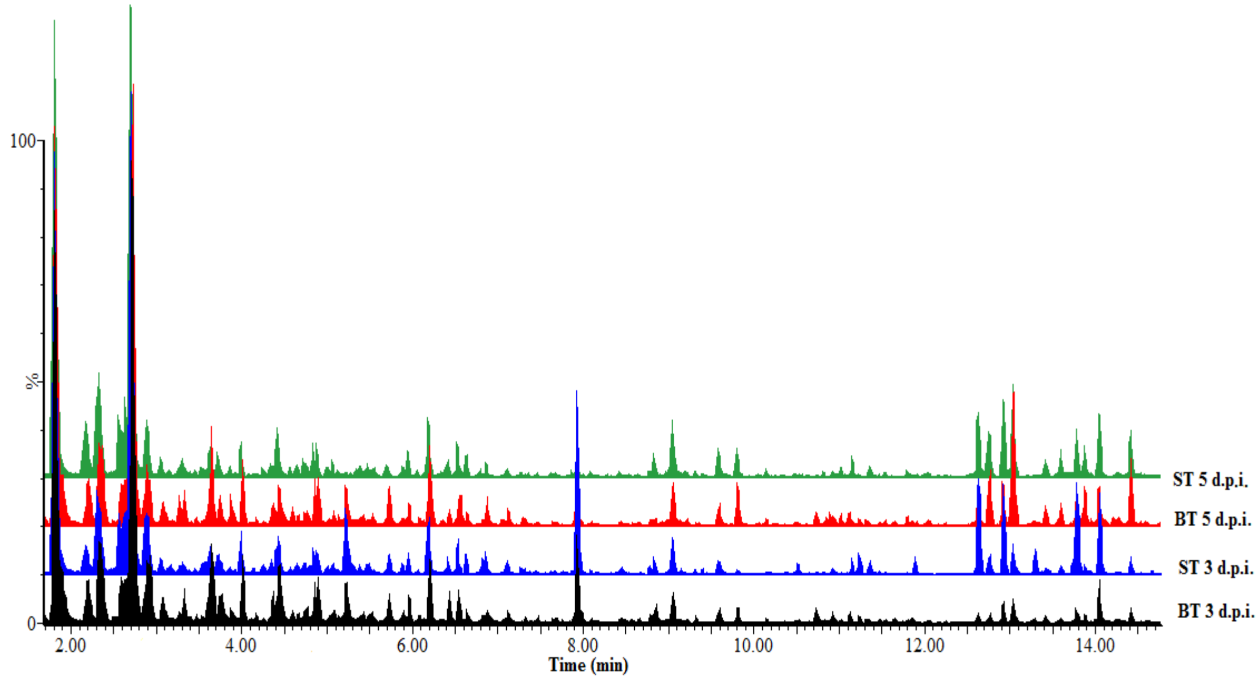

Figure S4: Comparative UHPLC-MS BPI chromatograms of extracts derived from sorghum NS 5511 (BT) *vs* NS 5655 (ST) cvs responding to infection by *B. andropogonis*. A: ESI negative and **B: ESI positive.** NS 5511-treated sample chromatograms at 3 and 5 d.p.i. shown against NS 5655-treated samples at the same time period, visually showing cultivar-related metabolic changes.

Note: For PCAs, to determine the group (control/non-infected) to which the time-related clusters in **(B)** belong, link to the corresponding positions in **(A)**. This applies to Figure S5 and Figure S6.

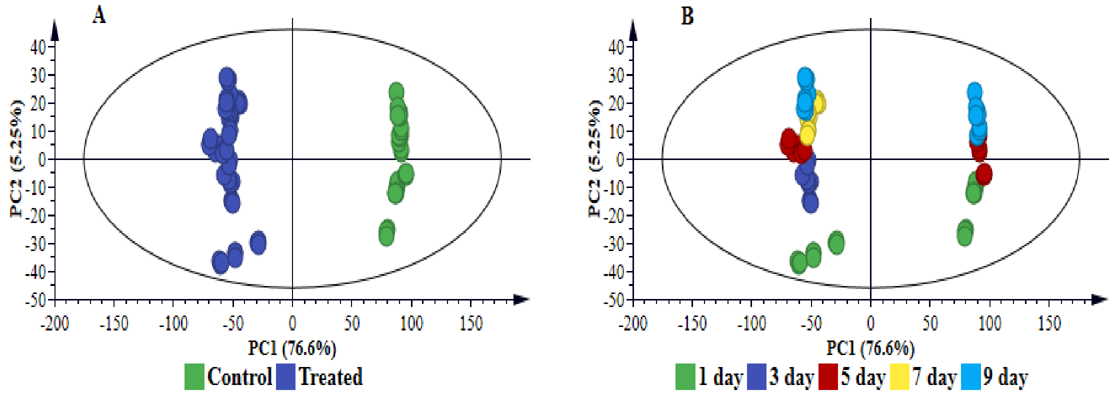

Figure S5: PC analyses of the ESI positive data for sorghum NS 5511 (BT) extracts. (A & B): A 10-component model, explaining 92.1% variations in Pareto-scaled data, X, and the amount of predicted variation by the model, according to cross-validation, is 86.7%. **(A)** and **(B)** represent the same scores plot, with (**A)** coloured according to treatment and (**B)** coloured according to time. This two-dimensional scores space, spanned by the first two PCs, reveals treatment-related sample clustering and also time-related clustering.

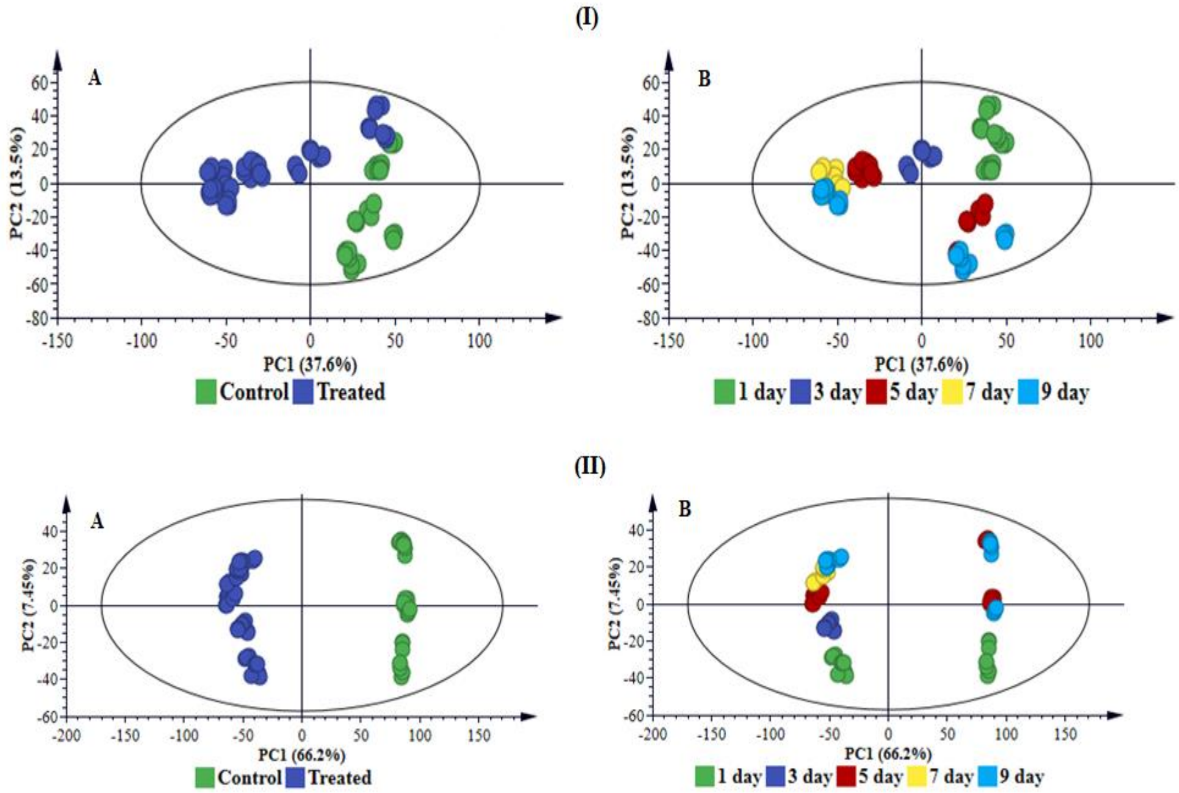

Figure S6: PC analyses of the (I) ESI negative and (II) ESI positive data for sorghum NS 5655 (ST) extracts. (I): A 8-component model, explaining 71.0% variations in Pareto-scaled data, X, and the amount of predicted variation by the model, according to cross-validation, is 60.4 %. **(II):** A 5-component model, explaining 82.0% variations in Pareto-scaled data, X, and the amount of predicted variation by the model, according to cross-validation, is 78.0%. The models labelled **(A)** and **(B)** represent the same scores plot, with one coloured according to treatment and the other according to time. These two-dimensional scores space, spanned by the first two PCs, reveals treatment-related sample clustering and also time-related clustering.

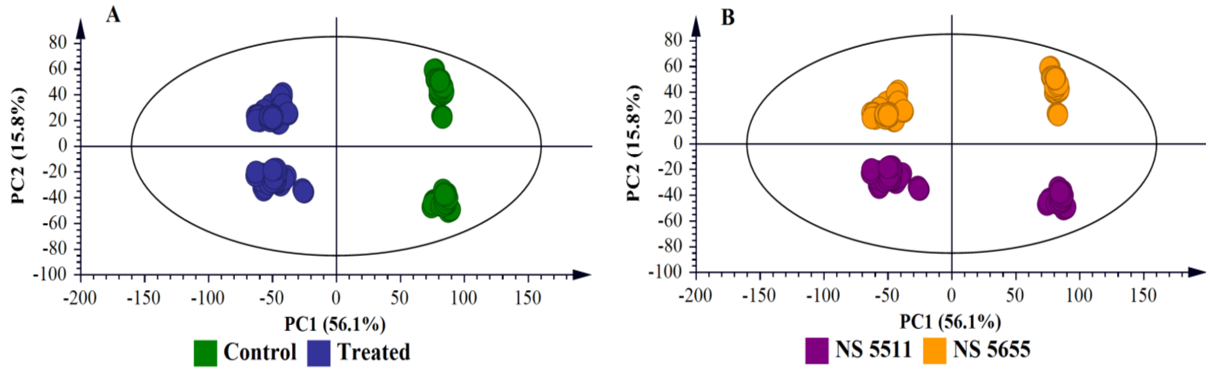

Figure S7: PC analyses of the <u>ESI positive</u> data for sorghum <u>NS 5511</u> (BT) and NS 5655 (ST) extracts. **(A** and **B):** A 14-component model, explaining 91.8% variation in Pareto-scaled data, X, and the amount of predicted variation by the model, according to cross-validation, is 87.7%. **(A)** and **(B)** represent the same scores plot, with **(A)** coloured according to treatment and **(B)** coloured according to cultivar. This two-dimensional scores space, spanned by the first two PCs, reveals treatment-related sample clustering and also cultivar-related clustering.

Another additional diagnostic tool used to validate OPLS-DA models was distance to the model in space X (DModX), for detecting moderate outliers. DModX plots **(Figures S8 A** and **S10 A)** show a few moderate outliers *i.e.* observations where the DModX value is above the Dcrit (critical value of DModX; 0.05). Moreover, permutation testing **(Figures S8 B** and **S10 B)**, as a powerful tool in measuring the performance of a model, was carried out to determine whether the performance of the computed binary classification (OPLS-DA) models were statistically significant. The permutation tests revealed that the computed OPLS-DA models were statistically significant and that in the separation of classes, none of the permutated models showed a better performance than that of the original models.

For further evaluation of the selected variables, dot plots (**Figures S8 C** and **S10 C**) were generated to investigate how discriminant the variables are. For instance, no overlap between the infected and control sample can be seen from the selected *m/z* = 327.21480 variable in **Figure S8 C**, also highlighted in the S-plot, and VIP plots, thus suggesting that the particular feature contributed strongly to the discrimination. **Note**: for model validation and evaluation of selected variables described in the main text and above, only figures for NS 5511 (BT) and NS 5655 (ST) in ESI negative ionisation mode are displayed. The same tools were used to for ESI positive data, however the data is not shown [17,19].

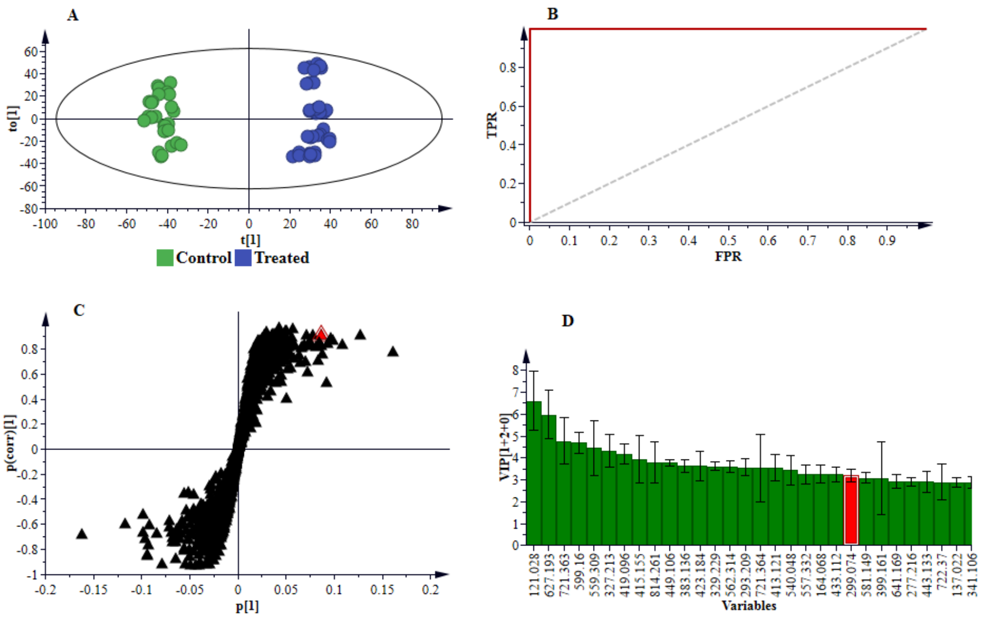

Figure S8: Supervised multivariate analyses of the <u>ESI negative</u> UHPLC-MS data for <u>NS 5655</u> (ST) cv extracts. (A): The OPLS-DA score plot shows grouping of control *vs* infected for all samples. This model comprises 1 predictive component and 2 orthogonal components (R^2X= 56.0%, R^2Y= 98.7% and Q^2= 97.6%). **(B):** A representative receiver operator characteristic (ROC) plot summarising the performance of OPLS-DA (a binary classification method). **(C):** OPLS-DA loading S-plot displays the discriminating features (ions) that explain the clustering (sample grouping) observed in the OPLS-DA scores plot, with the features in the top right quadrant positively correlated to the treatment and those in the bottom left quadrant negatively correlated to the treatment. This loading S-plot comprises 1 predictive component explaining 34.4% of the total variation and 2 orthogonal components explaining 22.0% of the total variation. **(D)**: A VIP plot summarising the importance of some of the variables in the projection of the model. A VIP value >1 is significant/important in the projection and higher score values indicate an increase in significance of the variables.

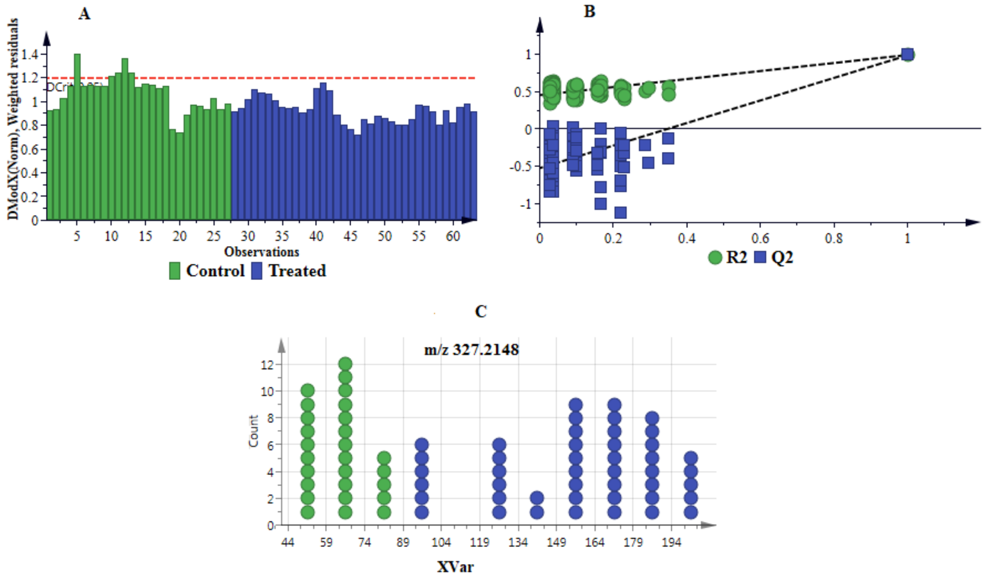

Figure S9: Supervised multivariate analyses of the <u>ESI negative</u> UHPLC-MS data for <u>NS 5511</u> (BT) cv extracts. (A): A distance to the model in space X (DModX) plot showing moderate outliers (above the dashed red line, Dcrit) in the OPLS-DA scores plot. **(B):** The response permutation test plot (n=100) of the OPLS-DA model, clearly separating control from infected plant samples. **(C):** Dot plot of the selected variable *m/z* **327.21480** from S-plot (in red; **Figure 3 C**, also marked on the VIP plot **Figure 3 D**) showing no overlap between control and infected groups, the variable thus strongly discriminates the two groups.

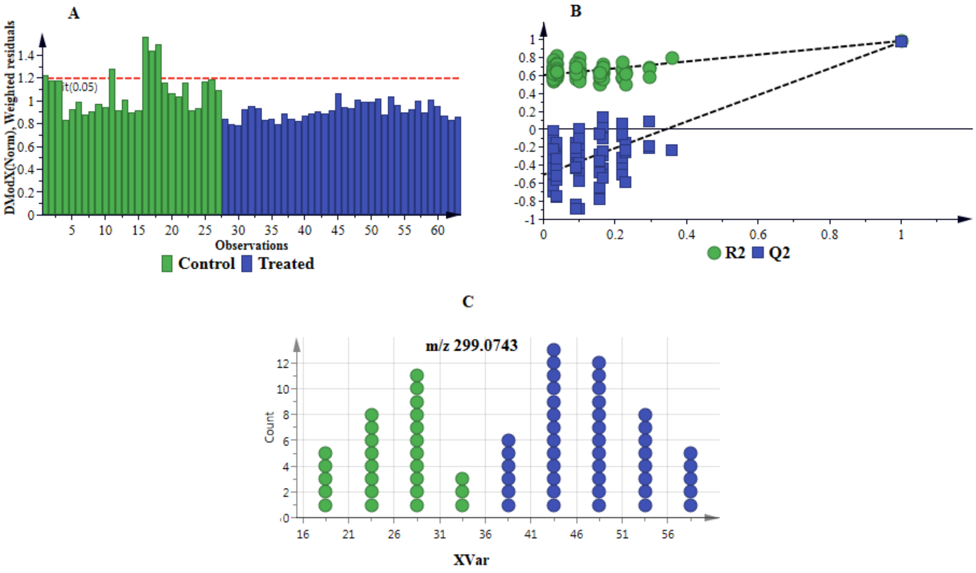

Figure S10: Supervised multivariate analyses of the <u>ESI negative</u> UHPLC-MS data for <u>NS 5655</u> (ST) cv extracts. (A): A distance to the model in space X (DModX) plot showing moderate outliers (above the dashed red line, Dcrit) in the OPLS-DA scores plot. **(B):** The response permutation test plot (n=100) of the OPLS-DA model, clearly separating control from infected plant samples. **(C):** Dot plot of the selected variable *m/z* **299.0743** from S-plot (in red; **Figure S9 C**, also marked on the VIP plot **Figure S9 D**) showing no overlap between control and infected groups, the variable thus strongly discriminates the two groups.

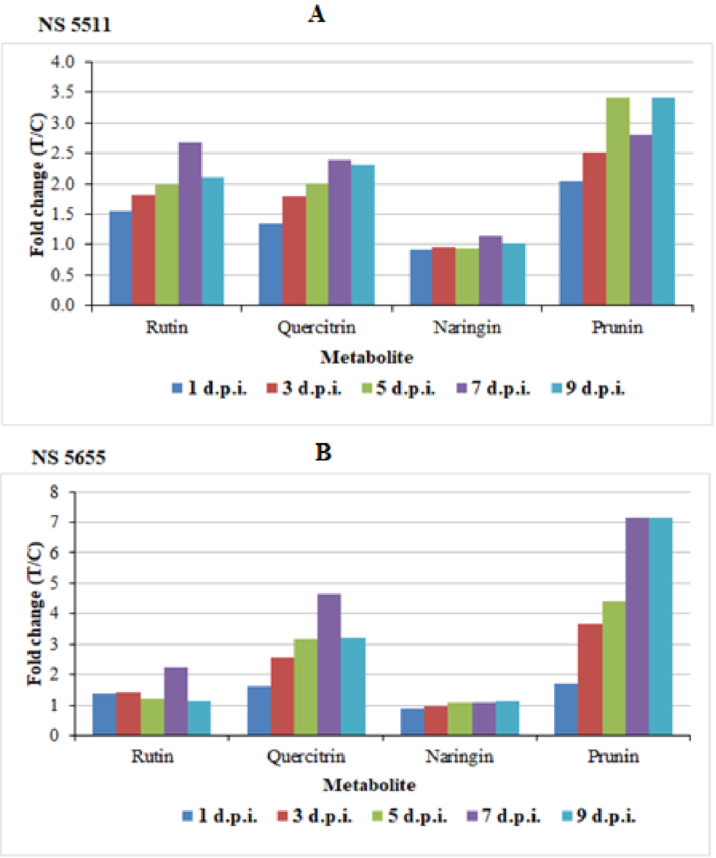

Figure S11: Relative quantification of flavanones and flavonols annotated in sorghum leaves responding to *B. andropogonis* infection. The relative levels of each metabolite are expressed in fold changes, computed from treated against control (T/C) samples, where fold change > 1 represents significant accumulation in **(A)** NS 5511 (BT) and **(B)** NS 5655 (ST). Rutin = quercetin rutinoside; quercitrin = quercetin 3-O-rhamnoside; naringin = naringenin 7-O-neohesperidoside; prunin = naringenin 7-O-beta-D-glucoside.

Table S2: Indicating diagnostic fragments for some of the metabolites annotated in Table 1.

m/z	Rt (min)	Ion mode	MS/MS Fragments	Metabolites
**269.007**	7.05	neg	225	Apigenin
**329.066**	8.92	neg	314, 299, 271, 163	Tricin
**431.099**	5.55	neg	415, 397, 379, 367, 341, 337, 311, 293, 283, 255	Apigenin-8-C-glucoside (vitexin)
**431.098**	6.33	neg	269	Apigetrin (apigenin 7-O-glucoside)
**433.114**	5.91	neg	271, 177, 151	Naringenin 7-O-beta-D-glucoside (prunin)
**447.091**	5.71	neg	285, 257, 175, 151	Luteolin 7-O-glucoside
**449.108**	4.57	neg	281, 167, 137, 123, 105	Pentahydroxychalcone 4'-O-glucoside
**563.139**	5.09	neg	443, 383, 353	Apigenin 8-C-xyloside-6-C-glucoside (vicenin-3)
**563.140**	4.87	neg	443, 383, 371, 353	Apigenin 6-C-xyloside-8-C-glucoside (vicenin-1)
**577.154**	5.32	neg	433,415, 367, 335, 293, 121	Vitexin 2''-O-rhamnoside
**577.156**	6.06	neg	269, 225	Apigenin 7-O-neohesperidoside (rhoifolin)
**593.150**	5.51	neg	285, 175	Luteolin 7-O-neohesperidoside
**593.151**	4.45	neg	473, 415, 383, 353	Apigenin-6,8-di-C-glucoside (vicenin-2)
**609.146**	5.43	neg	447,301, 255, 179, 151	Quercetin rutinoside (rutin)
**609.181**	4.80	neg	447, 433, 301, 281, 273, 163, 151, 107	Hesperidin
**625.180**	3.33	neg	271, 151, 119, 107	Naringenin 7-O-neohesperidoside (naringin)
**179.034**	4.35	neg	135	Caffeic acid
**193.048**	4.01	neg	178, 149, 134	Ferulic acid
**267.048**	4.38	neg	179, 135, 109	2-O-Caffeoylglyceric acid
**337.051**	3.29	neg	191, 173, 163, 119	4-Coumaroylquinic acid
**341.083**	6.17	neg	179, 162, 135	Caffeic acid hexose
**353.091**	3.58	neg	191, 179, 135	4-Caffeoylquinic acid
**355.102**	4.06	neg	193, 179, 134	1-O-Feruloyl-beta-D-glucose
**277.027**	2.63	neg	179, 135	4-Coumaroylagmatine
**367.099**	3.75	neg	193, 191, 134	3-Feruloylquinic acid
**371.062**	2.05	neg	191, 179, 135	2-O-Caffeoylglucarate
**413.121**	9.03	neg	193, 177, 163, 134, 121, 119	1,3-O-Coumaroyl-feruloylglycerol
**443.132**	9.22	neg	267, 249, 235, 207, 193, 161, 134	1,3-O-Diferuloylglycerol
**475.143**	1.92	neg	179, 161, 135	Caffeic acid derivative
**191.018**	1.10	neg	173, 155, 111	Isocitric acid
**311.220**	11.81	neg	293, 275, 235, 223	Dihydroxy-octadecadienoic acid
**327.215**	9.06	neg	291, 229, 208, 171	11,12,13-Trihydroxy-9,15-octadecadienoic acid
**299.074**	1.62	neg	137	Salicylic acid 2-O-beta-D-glucoside
**335.076**	4.64	neg	179, 174, 161, 135	Caffeoylshikimic acid
